# Supplementary material for: Synthesis of propenone-linked covalent organic frameworks via Claisen-Schmidt reaction for photocatalytic removal of uranium
Source: Nat Commun. 2023 Jul 21;14:4420. doi: 10.1038/s41467-023-40169-1 (PMC10361971; doi:10.1038/s41467-023-40169-1)
Supplement: Supplementary file 1 — Supplementary Information [file 41467_2023_40169_MOESM1_ESM.pdf]

Supplementary Information for

Synthesis of propenone-linked covalent organic frameworks via Claisen-Schmidt reaction for photocatalytic removal of uranium

Cheng-Peng Niu[1,†], Cheng-Rong Zhang[1,†], Xin Liu[1], Ru-Ping Liang[1]*, Jian-Ding Qiu[1,2]*

[1]School of Chemistry and Chemical Engineering, Nanchang University, Nanchang 330031, China

[2]State Key Laboratory of Nuclear Resources and Environment, East China University of Technology, Nanchang 330013, China

[†]These authors contributed equally.

*Corresponding authors: rpliang@ncu.edu.cn; jdqiu@ncu.edu.cn

Table of contents

1. Supplementary Notes.. 3

2. Supplementary Methods. 4

2.1 Synthesis of model compound...4

2.2 Electrochemical test...5

2.3 Adsorption experiment...6

2.4 Recyclability test...7

2.5 Antibacterial activity assay...8

2.6 DFT calculations..9

3. Supplementary Figures and Tables...10

4. Supplementary References...42

1. Supplementary Notes.

Sodium hydroxide, potassium hydroxide, acetic acid, aqua fortis, ascorbic acid, 1,4-dioxane, mesitylene, N,N-dimethylformamide (DMF), o-dichlorobenzene (O-DCB), piperidine, tetrahydrofuran (THF), propanone, ethanol and other nitrate salts were purchased from Sinopharm Chemical Reagent Co., Ltd. Trifluoroacetic acid, 1,8-Diazabicyclo[5,4,0]undec-7-ene (DBU), sodium ethoxide were purchased from Energy Chemical Technology (Shanghai) Co., Ltd. 1,3,6,8-Tetrakis-(4-formylphenyl)-pyrene (TFPPy), 5,5',5'',5'''-(pyrene-1,3,6,8-tetray)-tetrapicolinaldehyde (TFPPyN) were purchased from Jilin Chinese Academy of Sciences-Yanshen Technology Co., Ltd. 1,4-Diacetylbenzene (DAB), 4,4'-Diacetylbiphenyl (DBE) were purchased from Shanghai Aladdin Bio-Chem Technology Co., Ltd. *P. aeruginosa*, *S. aureus* and *V. alginolyticus* were purchased from China Center of Industrial Culture Collection. Water used in the experiment was prepared with a Millipore system (18.25 MΩ·cm). All reagents were used without further purification, and all the experiments were conducted at room temperature.

Fourier-transform infrared (FTIR) spectra were recorded with a Bruker TENSOR 27 instrument. Powder X-ray diffraction (PXRD) data of the nanomaterials were collected on a Bruker AXS D8 Advance A25 Powder X-ray diffractometer (40 kV, 40 mA) using Cu Kα (λ=1.5406 Å) radiation. X-ray photoelectron spectroscopy (XPS) spectra of the PyN-DAB and photocatalytic reduction products of U(VI) were performed on ground powders using a Thermo VG Multilab 2000X. Among them, the vacuum degree of the analysis chamber is 8^10-10 Pa, the excitation source adopts Al Kα rays (hν = 1486.6 eV), the working voltage is 12.5 kV, the filament current is 16 mA, and the signal accumulation is performed for 10 cycles. The test Passing-Energy full spectrum is 50 eV, the narrow spectrum is 20 eV, the step length is 0.05 eV, the residence time is 40-50 ms, and the charge correction is carried out with C 1s = 284.80 eV binding energy as the energy standard. The morphology of the material was imaged by a scanning electron microscope (SEM, JEM-2010, JEOL). The

morphology of the material was imaged by transmission electron microscopy (TEM, FEI Talos F200X G2, USA). The radiation stabilities of COFs were investigated in a GAMMATOR M-38-2 (USA) irradiator with a 60Co source (γ-ray). Solid-state ^{13}C cross-polarization magic-angle spinning (^{13}C CP/MAS NMR) spectra were recorded with a 4-mm double-resonance MAS probe; a sample spinning rate of 10.0 kHz, a contact time of 2 ms (ramp 100), and a pulse delay of 3 s were applied. The samples were outgassed at 120 ℃ for 12 h before the measurements. The nitrogen adsorption and desorption isotherms were measured at 77 K using a Micromeritics ASAP 2020M system. Surface areas were calculated from the adsorption data using Brunauer-Emmett-Teller (BET) methods. The pore-size-distribution curves were obtained via the non-local density functional theory (NLDFT) method. Metal ions concentrations were determined using an iCAP Q inductively coupled plasma mass spectrometry (ICP-MS, Thermo Fisher Scientific, USA). The thermal properties of the nanomaterials were evaluated using a STA PT1600 Linseis thermogravimetric analysis (TGA) instrument over the temperature range of 30 to 800 ℃ under nitrogen atmosphere with a heating rate of 10 ℃/min. UV-vis diffuse reflectance spectra (DRS) were recorded with a PE Lambda 900 UV/vis spectrophotometer at room temperature. Steady-state photoluminescence (PL) decay spectra were measured at room temperature using FLS 1000 spectrometer (Edinburgh Instruments, UK).

2. Supplementary Methods.

2.1 Synthesis of model compound

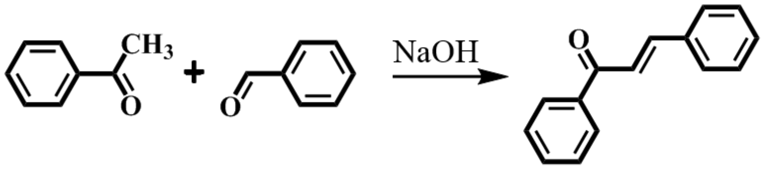

1,3-diphenyl-2-propenone was synthesized according to the reported procedure. To a 250 mL round bottom flask, 20 mmol of benzaldehyde and 20 mmol of acetophenone were added sequentially, 150 mL of water was used as the medium and 8 mmol of sodium hydroxide was used as the catalyst, the reaction was heated and

stirred until the end of the reaction (depending on the thin layer chromatography/TIC to determine if the reaction was complete and the conversion and selectivity of the reaction). At the end of the reaction, the reaction is left to stand and chilled until the product is precipitated. The solution is filtered through an extraction flask (water can be added during the extraction process) to obtain the crude product and the solid product is collected and dried. The fine product can be further separated and purified by column chromatography (δ 191.00, 144.94, 134.92, 132.89, 130.46, 128.47 (d, J = 9.8 Hz), 121.66.).

2.2 Electrochemical test

Indium-tin oxide (ITO) glasses were firstly cleaned by sonication in ethanol for 30 min and dried under nitrogen flow. 5 mg of COF powder was mixed with 1 mL *n*-BuOH and ultra-sonicated for 30 min to get slurry. 150 μL of the slurry was spreading onto ITO glass. After air drying, the boundary of the electrode was isolated with epoxy resin. A conventional three electrodes cell was used with a platinum mesh as the counter electrode and an Ag/AgCl electrode (saturated KCl) as reference electrode. The electrolyte was a 5 mM $K_3[Fe(CN)_6]$ aqueous solution and was purged with nitrogen gas for 1 h prior to the measurements. The working electrodes were immersed in the electrolyte for 60 s before any measurements were taken. The photocurrent responses were conducted with a CHI 760E workstation, with the working electrodes irradiated from the front side. The light was generated by a 300W xenon lamp (wavelength range 320 nm $\leq \lambda \leq$ 780 nm, light intensity 1 kW m^{-2}, Perfect Light, PLS-SXE300D) with a light density of 1 kW m^{-2} at room temperature with the light wavelength from 300 nm to 2500 nm. For Mott-Schottky experiments, the perturbation signal was 5 mV with the frequency of 1000, 2000 and 3000 Hz. Electrochemical impedance spectroscopy (EIS) measurements were performed in dark at open-circuit voltage of 5 mV with AC amplitude in the frequencies range of 0.01 Hz to 10^5 Hz. For CV experiments, the cyclic voltammograms were measured at a sweep rate at 100 mV s^{-1} and with voltage sweeps between 0 and -1.50 V.

2.3 Adsorption experiment

A stock solution of uranyl nitrate was prepared by dissolving appropriate amounts of $UO_2(NO_3)_2 \cdot 6H_2O$ in a suitable amount of nitric acid solution. The aqueous solutions of UO_2^{2+} with different concentrations were obtained by diluting the stock ions solution with the proper amount of ultra-pure water unless otherwise indicated. The pH levels of the solutions were adjusted by HNO_3 or NaOH aqueous solution. The concentrations of UO_2^{2+} during all the experiments were detected by inductively coupled plasma mass spectrometry (ICP-MS) for extra low concentrations. All adsorb experiments were carried out using 40 mL glass bottles at ambient temperature in air.

In general procedure, to obtain the uranium adsorption isotherms, 5 mg material was added into 35 mL solution of UO_2^{2+} with different concentrations in a bottle, respectively. The mixture was sonication for 30 seconds and shaken for 24 h at ambient temperature to achieve sorption equilibrium fully. Then the solid sorbent was filtered through a 0.22 μm membrane filter, followed by measuring the remaining uranium concentration using ICP-MS. The adsorbed amount at equilibrium (q_e, mg g^{-1}) was calculated by

$$q_e = \frac{(C_0 - C_e) \times V}{m} \tag{1}$$

where V is the volume of the treated solution (L), m is the amount of used adsorbent (g), and C_0 and C_e are the initial concentration and the final equilibrium concentration of uranium, respectively.

The equation of the Langmuir isotherm model was represented as following:

$$\frac{C_e}{q_e} = \frac{1}{q_m + k_L} + \frac{C_e}{q_m} \tag{2}$$

where q_m is the maximum adsorption when the adsorption reaches equilibrium, and k_L is a constant characterized by the affinity of the adsorbate with the adsorbent. The value of C_e/q_e as the function of C_e were plotted and fitted with a linear equation from which the q_m and k_L could be calculated according to the slope and intercept.

The Freundlich model is appropriate for multilayer sorption, which can be described as:

$$q_e = K_F C_e^n \quad (3)$$

where K_F (mg^{1-n} L^n/g) denotes the Freundlich sorption coefficient, and n expresses how favorable the sorption process is. K_F and n are empirical coefficients.

In the kinetics studies, 5 mg of the sorbent was added into a 35 mL solution containing 600 ppm uranium and used 0.1M HNO_3 to adjust the pH of the solution to 4.0. The mixture was stirred for a series of contact times, and the filtrate was collected at different contact time. The adsorption capacity of uranium as a function of contact time was obtained to determine the kinetics curve.

Pseudo-first-order kinetic model was described as the following function:

$$\ln(q_e - q_t) = \ln q_e - k_f t \quad (4)$$

where q_e represents the amount of U on the adsorbent under equilibrium, and k_f (min^{-1}) is pseudo-first-order adsorption rate constant.

Pseudo-second-order kinetic model was described as the following function:

$$\frac{t}{q_t} = \frac{1}{k_s + q_e^2} + \frac{1}{q_e} t \quad (5)$$

where q_e represents the amount of U on the adsorbent under equilibrium, and k_s (g mg^{-1} min^{-1}) is pseudo-second-order adsorption rate constant.

The anti-interference performance test of UO_2^{2+} adsorption from aqueous solution containing various metal ions was carried out at pH 4.0, the initial concentration of all competitive metal ions is 10 times of UO_2^{2+} and the residual concentration in the supernatant of metal ions was determined inductively coupled plasma mass spectrometry (ICP-MS).

2.4 Recyclability test

After one run of adsorption, the adsorbents were regenerated by treatment with the elution solution of 0.1 M HNO_3 solution and reused for another adsorption experiment. For 5 mg adsorbents, a certain amount of elution solution was used to elute the binding uranium at room temperature. The elution efficiency (E, %) was determined by using Equation:

$$E = \frac{C_e \times V_e}{(C_0 - C_t) \times V_t} \times 100\% \quad (6)$$

in where C_e (mg L^{-1}) is the uranium concentration in elution solution, V_e (L) is the volume elution solution, C_t (mg L^{-1}) is the uranium concentration in the simulated nuclear industry wastewater after uranium adsorption, C_0 (mg L^{-1}) is the initial uranium concentration of the simulated nuclear industry wastewater, V_t (L) is the volume of simulated nuclear industry wastewater used for adsorption. The resulting suspension was filtered and washed with ultra-pure water till the supernatant became neutral. After being dried under vacuum, the resultant material was used for another adsorption experiment. It was found that after five consecutive cycles still showed excellent uranium removal rate.

2.5 Antibacterial activity assay

Pseudomonas aeruginosa strain CICC 10205 (*P. aeruginosa*), Staphylococcus aureus strain CICC 10001 (*S. aureus*), Vibrio alginolyticus strain CICC 21664 (*V. alginolyticus*) were used to test the antimicrobial spectrum of the adsorbents. The exponential growth bacteria and the adsorbents were transferred into fresh LB broth at a ratio of 1% (V/V) and 0.05% (m/V), respectively. After cultivated at 37 ℃ for 4 h with moderate shaking (180 rpm), the viability of the bacterium was determined. The dilution plate counting method was used according to the Chinese standard GB/T20944 to determine the viability of the adsorbents treated bacterial cultures. In brief, sterilized LB solid medium was poured into the aseptic plates to prepare sterile plate count agar plates under aseptic conditions and bacterial cultures were spread onto the plate after gradient dilution. After cultivating overnight at 37 ℃, the number of viable bacterial cells was determined by counting the bacterial clones formed on the plate. The bacterial cultures without treating by adsorbent were used as control. The inhibition rate was calculated using the Equation:

$$\mathrm{IR} = \left(1 - \frac{C_a}{C_i}\right) \times 100\% \quad (7)$$

in where C_a (CFU/mL) indicates the concentration of bacterial cultures treated with adsorbent and C_i (CFU/mL) indicates the concentration of bacterial cultures without treatment. The simulated sunlight with a light density of 1 kW m^{-2} was used

to illuminate the adsorbents.

2.6 DFT calculations

The ground state geometry is optimized using DFT. All calculations are performed with the Gaussian 16 package (Rev. C.01) using the hybrid B3LYP functional and the 6-311G(d)/SDD basis set. Grimme's D3BJ dispersion correction was used to improve calculation accuracy. The D index formula for measuring the distance between holes and the center of mass of electrons:

$$D_x = |X_{ele} - X_{hole}| \quad D_y = |Y_{ele} - Y_{hole}| \quad D_z = |Z_{ele} - Z_{hole}| \tag{8}$$

$$D \text{ index} = \sqrt{(D_X)^2 + (D_y)^2 + (D_z)^2} \tag{9}$$

3. Supplementary Figures and Tables.

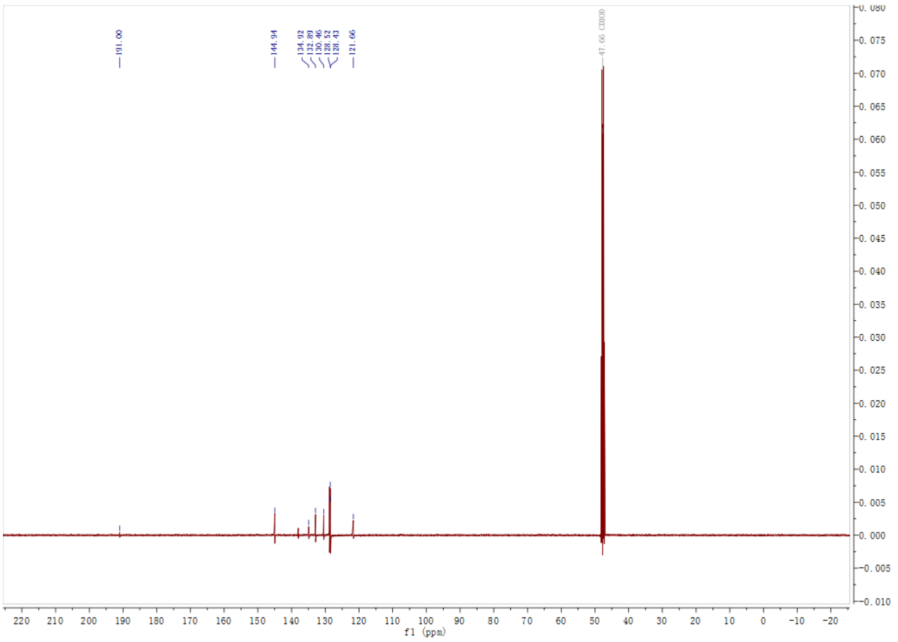

Supplementary Fig. 1 | ^{13}C NMR spectrum of 1,3-diphenyl-2-propenone recorded in CD_3OD.

Supplementary Table 1 | Synthesis of the Py-DAB under variable conditions.

Scheme	Solvent	Temp	Reaction Times	degree of crystallinity
1	1, 4-dioxane/KOH	90°C	3d	High
2	1, 4-dioxane /mesitylene/NaOH	120°C	3d	Medium
3	O-DCB/n-BuOH/KOH	150°C	3d	Low
4	1, 4-dioxane/95% ethanol/NaOH	120°C	3d	Medium
5	mesitylene/TFA/1, 4-dioxane/acetonitrile	150°C	3d	No
6	O-DCB/n-BuOH/EtONa	120°C	3d	No
7	methanol/EtONa/ O-DCB	40/90/ 110°C	6/48/24h	No
8	O-DCB/DBU	90°C	3d	No product
9	dimethylamine(THF) /O-DCB/DMF	180°C	3d	No product
10	piperidine/DMF	180°C	3d	No product

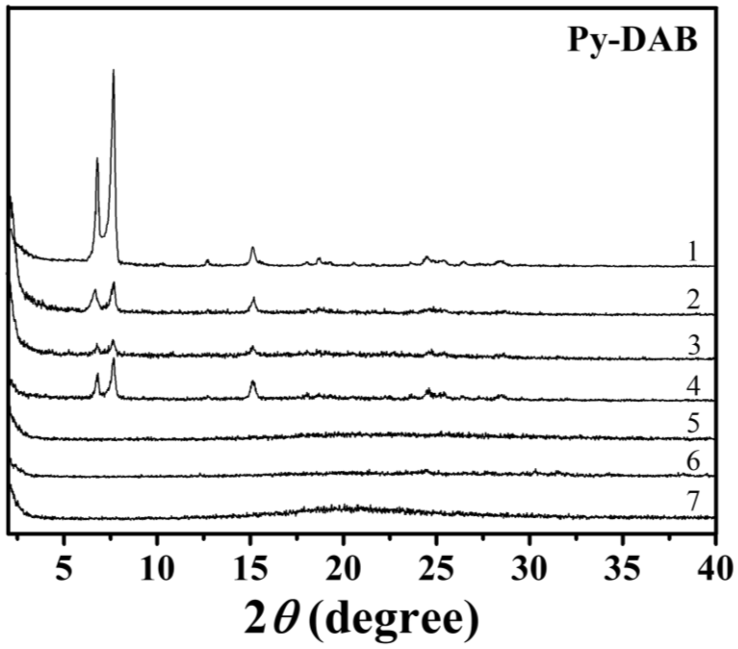

Supplementary Fig. 2 | PXRD patterns of attempts to synthesize Py-DAB under above experimental scheme.

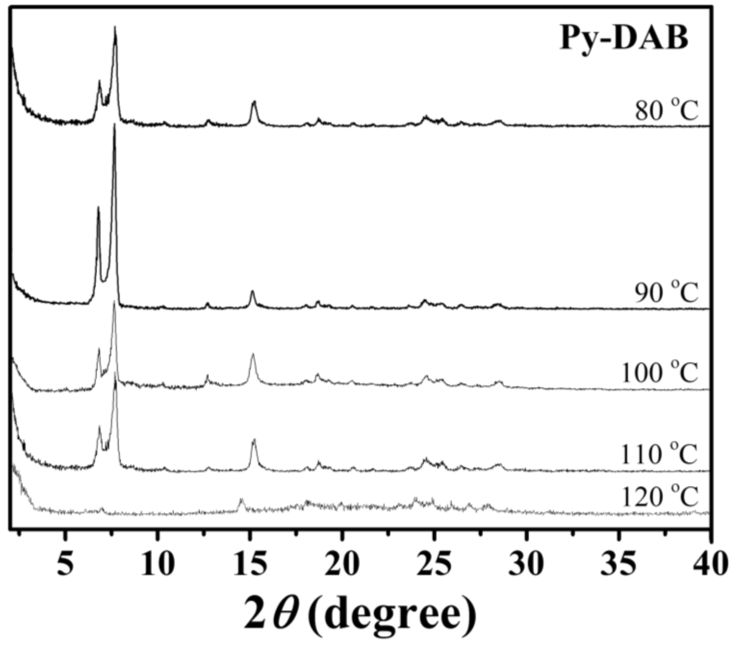

Supplementary Fig. 3 | PXRD patterns of attempts to synthesize Py-DAB under the condition of scheme 1 with temperature optimization.

Supplementary Table 2 | Synthesis of the PyN-DAB under variable conditions.

Scheme	Solvent	Temp	Reaction Times	degree of crystallinity
1	1, 4-dioxane/KOH	90℃	3d	High
2	1, 4-dioxane /mesitylene/NaOH	120℃	3d	Low
3	O-DCB/n-BuOH/KOH	150℃	3d	No
4	1, 4-dioxane/95% ethanol/NaOH	120℃	3d	No
5	mesitylene/TFA/1, 4-dioxane/acetonitrile	150℃	3d	No
6	O-DCB/n-BuOH/EtONa	120℃	3d	No
7	methanol/EtONa/ O-DCB	40/90/ 110℃	6/48/24h	No
8	O-DCB/DBU	90℃	3d	No product
9	dimethylamine(THF) /O-DCB/DMF	180℃	3d	No product
10	piperidine/DMF	180℃	3d	No product

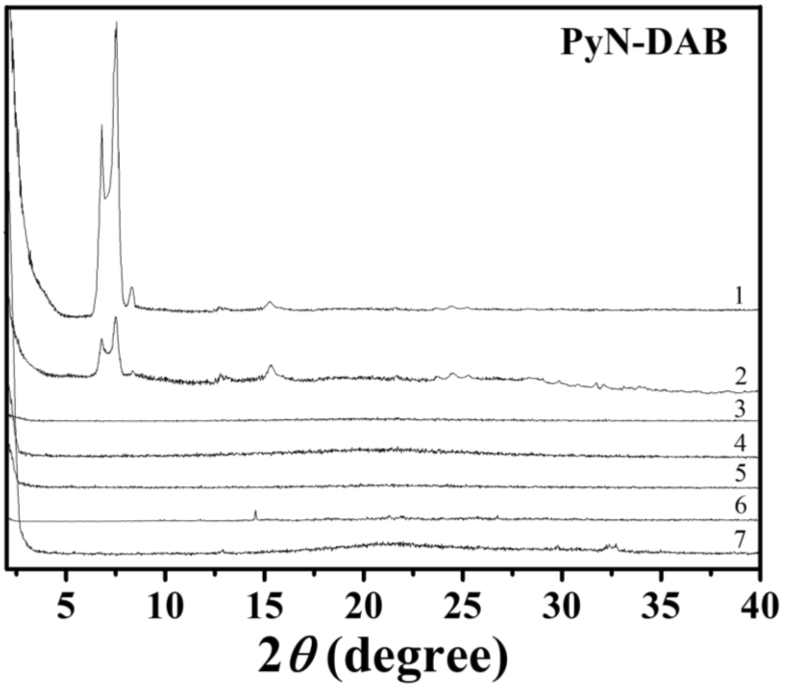

Supplementary Fig. 4 | PXRD patterns of attempts to synthesize PyN-DAB under above experimental scheme.

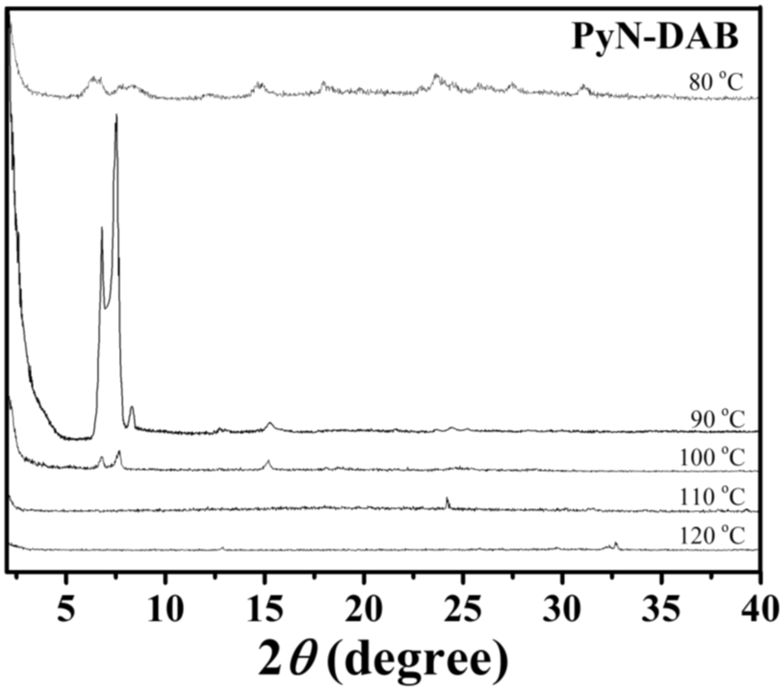

Supplementary Fig. 5 | PXRD patterns of attempts to synthesize PyN-DAB under the condition of scheme 1 with temperature optimization.

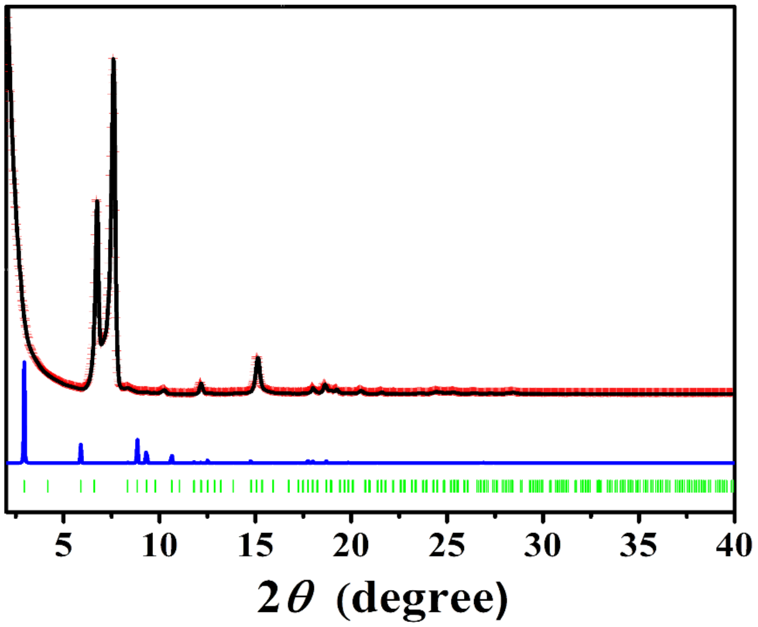

Supplementary Fig. 6 | PXRD patterns of Py-DAB with the experimental (red cross) and Pawley refined (black line) profiles, the refinement difference (orange line), the eclipsed AA stacking model (blue line) and the Bragg position (green bar).

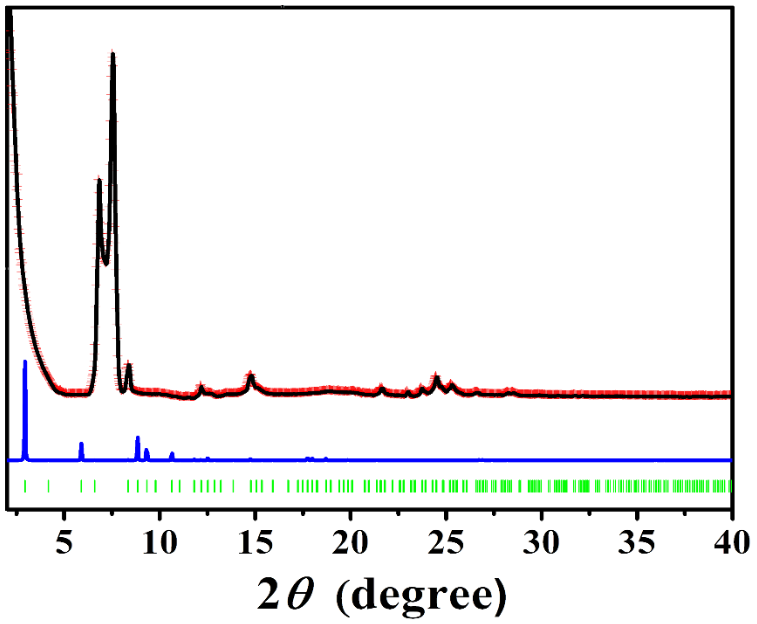

Supplementary Fig. 7 | PXRD patterns of PyN-DAB with the experimental (red cross) and Pawley refined (black line) profiles, the refinement difference (orange line), the eclipsed AA stacking model (blue line) and the Bragg position (green bar).

Supplementary Table 3 | Fractional atomic coordinates for the eclipsed AB-stacking unit cell of Py-DAB.

Py-DAB (P1)<br>a=26.2038 Å, b=26.3714 Å, c=13.0242 Å<br>α=90°, β=90°, γ=90°							
**Atom**	**x(Å)**	**y(Å)**	**z(Å)**	**Atom**	**x(Å)**	**y(Å)**	**z(Å)**
**C1**	0.59689	0.40877	0.46118	**C107**	0.0035	0.93298	0.96539
**C2**	0.61032	0.46044	0.46126	**C108**	0.0412	0.89462	0.96452
**C3**	0.57073	0.49714	0.45452	**C109**	0.95111	0.92155	0.95942
**C4**	0.51898	0.48206	0.4631	**C110**	0.91466	0.95984	0.95862
**C5**	0.50643	0.43013	0.46782	**C111**	0.92892	0.01117	0.96591
**C6**	0.54563	0.3934	0.45982	**C112**	0.98108	0.02328	0.9728
**C7**	1.4549	0.41626	0.4795	**C113**	0.08357	0.04815	0.97494
**C8**	1.4167	0.4528	0.48492	**C114**	0.0473	0.08381	1.00481
**C9**	1.42822	-0.49511	0.47903	**C115**	0.99627	0.07445	0.98167
**C10**	1.47959	-0.48063	0.46489	**C116**	0.96086	0.11442	0.96207
**C11**	0.58044	-0.45146	0.43983	**C117**	0.90856	0.10118	0.95766
**C12**	0.54352	-0.41665	0.43767	**C118**	0.8924	0.05063	0.96619
**C13**	1.49199	-0.42901	0.45031	**C119**	0.83735	0.03915	0.97633
**C14**	1.45321	-0.39188	0.44615	**C120**	0.97724	0.16773	0.93069
**C15**	1.40251	-0.40624	0.4665	**C121**	0.16178	0.96913	1.03333
**C16**	1.38973	-0.45728	0.48515	**C122**	0.02737	0.84116	0.94379
**C17**	1.33626	-0.47086	0.51123	**C123**	0.0245	0.1772	0.88182
**C18**	1.46553	-0.33845	0.41895	**C124**	0.03798	0.22523	0.84595
**C19**	0.66478	0.47462	0.48151	**C125**	0.00438	0.26609	0.85731
**C20**	0.53311	0.3392	0.44498	**C126**	0.95707	0.25773	0.90556
**C21**	0.49354	-0.32814	0.3295	**C127**	0.94398	0.20942	0.94227
**C22**	0.5127	-0.2797	0.3108	**C128**	0.82055	1.01139	1.06184
**C23**	0.50295	-0.23988	0.37935	**C129**	0.77198	0.98964	1.06227
**C24**	1.47273	-0.24952	0.46684	**C130**	0.7389	0.99752	0.97947
**C25**	1.45419	-0.29852	0.48633	**C131**	0.75388	0.02916	0.8981
**C26**	1.32606	0.5019	0.60162	**C132**	0.80317	0.04978	0.89633
**C27**	1.27848	0.47907	0.61652	**C133**	0.03164	0.80427	1.02088
**C28**	1.23953	0.48542	0.54392	**C134**	0.01022	0.75611	1.00647
**C29**	1.24837	-0.48376	0.45748	**C135**	0.98399	0.74462	0.91512
**C30**	1.29657	-0.46217	0.4411	**C136**	0.98176	0.78094	0.83665
**C31**	0.54281	0.30349	0.5223	**C137**	1.00411	0.82852	0.85022

**C32**	0.5227	0.25436	0.51456	**C138**	0.20285	0.9368	1.00875
**C33**	1.49253	0.24076	0.42939	**C139**	0.25165	0.94487	1.05003
**C34**	1.48494	0.276	0.35034	**C140**	0.26075	0.98584	1.1167
**C35**	1.50557	0.32463	0.35746	**C141**	0.22044	0.01838	1.14154
**C36**	0.69024	0.45408	0.56718	**C142**	0.17196	0.01007	1.1004
**C37**	0.73924	0.4709	0.59434	**C143**	0.69231	0.96689	0.9721
**C38**	0.76334	0.50892	0.53636	**C144**	0.64571	-0.01256	0.97165
**C39**	0.73858	-0.47166	0.44983	**C145**	0.60004	0.95489	0.96343
**C40**	0.69077	-0.48926	0.42173	**C146**	0.55023	0.96892	1.01001
**C41**	1.19203	0.45574	0.55491	**O147**	0.60363	0.91493	0.91679
**C42**	1.14606	-0.52576	0.53051	**C148**	0.02139	0.31671	0.8215
**C43**	1.09968	0.44271	0.53926	**C149**	1.00437	0.36078	0.86011
**C44**	1.04884	0.46529	0.56067	**C150**	0.02746	0.40982	0.83002
**O45**	1.1034	0.39699	0.5272	**C151**	0.00501	0.4591	0.86132
**C46**	0.52813	-0.19035	0.36039	**O152**	0.06693	0.40998	0.78022
**C47**	1.51154	-0.1459	0.39802	**C153**	0.50615	0.94158	0.98307
**C48**	0.53861	-0.09765	0.37604	**C154**	0.45902	0.95279	1.02804
**C49**	0.51648	-0.04721	0.40197	**C155**	0.4548	0.99178	1.10137
**O50**	0.5806	-0.09925	0.33548	**C156**	0.49912	0.01853	1.12952
**C51**	1.00431	0.43735	0.54026	**C157**	0.54626	0.00734	1.08454
**C52**	0.95606	0.45754	0.56183	**C158**	0.03661	0.50199	0.86799
**C53**	0.9513	0.50612	0.60583	**C159**	0.01628	0.54964	0.89098
**C54**	0.996	-0.46616	0.62659	**C160**	0.96355	0.55548	0.90808
**C55**	1.04412	-0.48623	0.60454	**C161**	0.93196	0.51241	0.90249
**C56**	0.54831	-0.00427	0.40427	**C162**	0.95242	0.46469	0.87956
**C57**	0.52831	0.04402	0.42319	**C163**	0.95508	0.69699	0.90272
**C58**	1.47573	0.05053	0.44103	**C164**	0.9713	0.6524	0.93935
**C59**	1.44398	0.00755	0.43952	**C165**	0.94028	0.60618	0.92564
**C60**	1.46392	-0.04064	0.41975	**O166**	0.89393	0.61017	0.92755
**C61**	1.46458	0.19224	0.42486	**C167**	0.31177	-0.00312	1.1597
**C62**	1.48316	0.14845	0.461	**C168**	0.35546	0.98864	1.1087
**C63**	1.45247	0.10156	0.45472	**C169**	0.40539	0.00436	1.15168
**O64**	1.40612	0.10521	0.45958	**O170**	0.406	0.02944	1.23049
**C65**	0.81217	-0.46815	0.56821	**H171**	0.11946	0.87678	0.99297
**C66**	0.85185	0.5047	0.6024	**H172**	0.93747	0.88289	0.95631
**C67**	0.90077	-0.47023	0.62737	**H173**	0.8751	0.94853	0.95163
**O68**	0.89955	-0.42782	0.66483	**H174**	0.12346	0.05818	0.96149
**H69**	0.62685	0.38048	0.45988	**H175**	0.05749	0.12208	1.02951

**H70**	1.44352	0.37695	0.48588	**H176**	0.8798	0.12981	0.94564
**H71**	1.37789	0.4394	0.49239	**H177**	0.05562	0.14771	0.89689
**H72**	0.61702	-0.43574	0.41235	**H178**	0.07608	0.23139	0.81535
**H73**	0.56116	-0.38205	0.41901	**H179**	0.93	0.28819	0.91517
**H74**	1.37295	-0.37755	0.46701	**H180**	0.90817	0.20531	0.98223
**H75**	0.50535	-0.35915	0.28017	**H181**	0.84711	1.00311	1.1236
**H76**	1.46622	-0.22042	0.52368	**H182**	0.76097	0.96486	1.12496
**H77**	1.43389	-0.30609	0.55709	**H183**	0.72882	0.03486	0.83295
**H78**	1.35667	0.49487	0.65591	**H184**	0.81587	0.07105	0.82985
**H79**	1.21911	-0.47908	0.3995	**H185**	0.04915	0.81375	1.09374
**H80**	1.30388	-0.44146	0.37078	**H186**	0.01149	0.72905	1.069
**H81**	0.56365	0.31455	0.59061	**H187**	0.96159	0.77293	0.76582
**H82**	0.52813	0.22817	0.57766	**H188**	1.0008	0.85677	0.79
**H83**	1.49795	0.35203	0.29724	**H189**	0.19765	0.90587	0.95545
**H84**	0.67111	0.42673	0.61559	**H190**	0.28205	0.91911	1.02964
**H85**	0.75693	0.45629	0.66321	**H191**	0.22758	0.05232	1.18674
**H86**	0.67364	-0.47101	0.35683	**H192**	0.14381	0.04311	1.0944
**H87**	1.19474	0.41753	0.58605	**H193**	0.6966	0.92587	0.97056
**H88**	1.14221	-0.48689	0.50513	**H194**	0.64125	0.02809	0.97884
**H89**	0.56163	-0.19063	0.31118	**H195**	0.05218	0.31749	0.76561
**H90**	1.47773	-0.14497	0.44517	**H196**	0.97557	0.36094	0.91941
**H91**	1.00721	0.39977	0.50683	**H197**	0.5086	0.91097	0.9275
**H92**	0.92286	0.43502	0.54298	**H198**	0.42644	0.92992	1.00684
**H93**	0.99345	-0.42864	0.66031	**H199**	0.49704	0.0481	1.18723
**H94**	1.07751	-0.46412	0.62398	**H200**	0.57958	0.0281	1.10994
**H95**	0.58889	-0.00837	0.38995	**H201**	0.07719	0.49837	0.8534
**H96**	0.55408	0.07602	0.42126	**H202**	0.04187	0.58183	0.8923
**H97**	1.40303	0.01189	0.44911	**H203**	0.89102	0.51632	0.91364
**H98**	1.43755	-0.07209	0.4146	**H204**	0.92677	0.43267	0.87284
**H99**	1.4275	0.19224	0.38721	**H205**	0.91971	0.69828	0.85897
**H100**	0.52104	0.14698	0.49373	**H206**	0.00776	0.64968	0.97787
**H101**	0.81652	-0.42761	0.55652	**H207**	0.31299	0.01621	1.23325
**H102**	0.84873	0.46394	0.60769	**H208**	0.35398	0.97292	1.03217
**C103**	0.09234	0.90727	0.98382	**H209**	0.27254	1.45503	0.68308
**C104**	0.10828	0.95825	0.99384	**H210**	0.46184	1.26631	0.2841
**C105**	0.07079	0.99679	0.97904	**H211**	0.53756	1.72566	0.24527
**C106**	0.01847	0.98415	0.97326	**H212**	0.75521	1.55801	0.40352

Supplementary Table 4 | Fractional atomic coordinates for the eclipsed AB-stacking unit cell of PyN-DAB.

**PyN-DAB (P1)<br>a=26.1833 Å, b=26.3842 Å, c=13.0745 Å<br>α=90°, β=90°, γ=90 °**							
**Atom**	**x(Å)**	**y(Å)**	**z(Å)**	**Atom**	**x(Å)**	**y(Å)**	**z(Å)**
**C1**	0.59677	0.40874	0.46062	**C105**	0.07106	0.99684	0.97714
**C2**	0.61028	0.46037	0.46081	**C106**	0.01864	0.98414	0.97303
**C3**	0.57073	0.49714	0.45459	**C107**	0.00368	0.93296	0.96722
**C4**	0.51892	0.4821	0.46287	**C108**	0.04146	0.89467	0.96698
**C5**	0.50627	0.43019	0.46712	**C109**	0.95123	0.92142	0.96275
**C6**	0.54546	0.39343	0.45927	**C110**	0.91468	0.95966	0.96173
**C7**	1.45468	0.41639	0.47839	**C111**	0.92891	0.01105	0.96708
**C8**	1.41652	0.45297	0.48414	**C112**	0.98117	0.02322	0.97253
**C9**	1.42816	-0.49498	0.47858	**C113**	0.08374	0.04812	0.97094
**C10**	1.47957	-0.48055	0.46477	**C114**	0.0477	0.08374	1.00185
**C11**	0.58054	-0.45143	0.4407	**C115**	0.99645	0.07437	0.98032
**C12**	0.54367	-0.41657	0.43885	**C116**	0.96095	0.11432	0.96147
**C13**	1.49206	-0.42894	0.45074	**C117**	0.90855	0.10111	0.95811
**C14**	1.45329	-0.39179	0.44618	**C118**	0.89233	0.05054	0.96694
**C15**	1.40252	-0.4061	0.46609	**C119**	0.8371	0.03906	0.97542
**C16**	1.38972	-0.45711	0.48474	**C120**	0.97736	0.16766	0.93023
**C17**	1.3362	-0.47062	0.51046	**C121**	0.16228	0.9697	1.03189
**C18**	1.46566	-0.33842	0.41891	**C122**	0.02768	0.84121	0.9468
**C19**	0.66484	0.47437	0.48041	**C123**	0.02437	0.17713	0.88039
**C20**	0.53301	0.3392	0.44486	**C124**	0.03808	0.22528	0.84582
**C21**	0.49393	-0.32819	0.33013	**C125**	0.00485	0.26632	0.85892
**N22**	0.51305	-0.27978	0.31138	**C126**	0.95777	0.25797	0.90812
**C23**	0.5026	-0.23978	0.3788	**C127**	0.94452	0.20952	0.94377
**C24**	1.47217	-0.24936	0.46568	**C128**	0.81926	1.01218	1.06096
**C25**	1.45397	-0.29841	0.48552	**C129**	0.77045	0.99077	1.0604
**C26**	1.32581	0.50257	0.60097	**C130**	0.7383	0.99782	0.97616
**N27**	1.27799	0.48023	0.6163	**C131**	0.75428	0.02848	0.89446
**C28**	1.23921	0.48604	0.54339	**C132**	0.80372	0.04886	0.89385
**C29**	1.2484	-0.48394	0.45626	**C133**	0.03127	0.80446	1.02393
**C30**	1.29666	-0.46236	0.44003	**C134**	0.0098	0.75632	1.00921
**C31**	0.54279	0.30362	0.52202	**C135**	0.98449	0.74473	0.91712

**C32**	0.52324	0.2543	0.51405	**C136**	0.98316	0.78087	0.83868
**C33**	1.49359	0.24031	0.42872	**C137**	1.00521	0.82849	0.85282
**N34**	1.48592	0.27545	0.34985	**C138**	0.20325	0.93673	1.00993
**C35**	1.50603	0.32429	0.35719	**C139**	0.25185	0.94474	1.05212
**C36**	0.69063	0.45347	0.565	**C140**	0.26099	0.98641	1.11668
**C37**	0.73967	0.47032	0.5918	**C141**	0.2209	0.01978	1.13831
**C38**	0.76348	0.50868	0.53446	**C142**	0.17254	0.01143	1.09657
**N39**	0.73855	-0.47185	0.44856	**C143**	0.69177	0.96719	0.96785
**C40**	0.69068	-0.48943	0.42087	**C144**	0.64511	-0.01242	0.96998
**C41**	1.19165	0.45647	0.55502	**C145**	0.5995	0.95496	0.96195
**C42**	1.14567	-0.52561	0.52886	**C146**	0.55005	0.96821	1.01088
**C43**	1.09942	0.44274	0.53943	**O147**	0.60285	0.9156	0.91341
**C44**	1.04855	0.46519	0.56157	**C148**	0.02219	0.31702	0.82398
**O45**	1.10332	0.39699	0.52859	**C149**	1.00374	0.3612	0.85921
**C46**	0.52744	-0.19018	0.35936	**C150**	0.02706	0.41017	0.82962
**C47**	1.51103	-0.14584	0.39746	**C151**	0.0042	0.45946	0.85931
**C48**	0.53786	-0.09759	0.37483	**O152**	0.06724	0.41029	0.78218
**C49**	0.51616	-0.04733	0.40286	**C153**	0.50618	0.93987	0.98692
**O50**	0.57931	-0.099	0.33207	**C154**	0.45929	0.95066	1.03317
**C51**	1.00402	0.43651	0.5455	**C155**	0.45508	0.99026	1.10481
**C52**	0.95579	0.45681	0.56696	**C156**	0.49925	0.01782	1.13055
**C53**	0.95106	0.50625	0.60661	**C157**	0.54619	0.007	1.08437
**C54**	0.99576	-0.46542	0.62379	**C158**	0.03593	0.50211	0.86817
**C55**	1.04384	-0.48559	0.60171	**C159**	0.01561	0.54973	0.89086
**C56**	0.54833	-0.00466	0.40586	**C160**	0.96271	0.55585	0.90551
**C57**	0.52868	0.04361	0.42592	**C161**	0.93086	0.51304	0.8972
**C58**	1.47615	0.05036	0.44421	**C162**	0.95133	0.46528	0.8745
**C59**	1.4441	0.0076	0.44261	**C163**	0.9552	0.69742	0.90402
**C60**	1.46368	-0.04058	0.42162	**C164**	0.97096	0.65251	0.93947
**C61**	1.46592	0.19162	0.42425	**C165**	0.93968	0.6066	0.92354
**C62**	1.48412	0.1482	0.4626	**O166**	0.89332	0.61091	0.92376
**C63**	1.45319	0.10147	0.45777	**C167**	0.31183	-0.00283	1.1608
**O64**	1.40685	0.10538	0.46324	**C168**	0.35579	0.98743	1.11198
**C65**	0.81223	-0.46818	0.5662	**C169**	0.40575	0.0028	1.15527
**C66**	0.85164	0.50496	0.60237	**O170**	0.40647	0.02761	1.23411
**C67**	0.90053	-0.46985	0.62697	**H171**	0.11982	0.87702	0.99515
**O68**	0.89928	-0.4271	0.66264	**H172**	0.93774	0.88269	0.96114

**H69**	0.62673	0.38042	0.45938	**H173**	0.87507	0.94819	0.95642
**H70**	1.44321	0.37711	0.48424	**H174**	0.12356	0.05813	0.95628
**H71**	1.37763	0.4397	0.49169	**H175**	0.05816	0.12195	1.0262
**H72**	0.61716	-0.43569	0.41347	**H176**	0.87976	0.12984	0.94666
**H73**	0.56137	-0.38195	0.42078	**H177**	0.05547	0.14747	0.89402
**H74**	1.37291	-0.37741	0.46627	**H178**	0.0761	0.2313	0.81462
**H75**	0.50594	-0.35923	0.28139	**H179**	0.931	0.28854	0.91961
**H76**	1.46507	-0.22014	0.52174	**H180**	0.90895	0.20544	0.98453
**H77**	1.43365	-0.30593	0.55592	**H181**	0.84514	1.00436	1.12387
**H78**	1.3564	0.49557	0.65519	**H182**	0.75855	0.9668	1.12346
**H79**	1.2194	-0.47994	0.39781	**H183**	0.72983	0.0336	0.8284
**H80**	1.30415	-0.44203	0.36955	**H184**	0.81716	0.06928	0.82716
**H81**	0.5632	0.31491	0.59046	**H185**	0.04812	0.81408	1.09714
**H82**	0.52873	0.22822	0.57699	**H186**	0.01025	0.7294	1.07171
**H83**	1.49848	0.35156	0.29698	**H187**	0.96382	0.77272	0.76721
**H84**	0.67174	0.42588	0.61302	**H188**	1.00228	0.85671	0.79279
**H85**	0.75763	0.45543	0.65987	**H189**	0.19809	0.90512	0.95844
**H86**	0.67333	-0.47104	0.35665	**H190**	0.28207	0.91837	1.03415
**H87**	1.19436	0.41882	0.58859	**H191**	0.2281	0.05415	1.18182
**H88**	1.14174	-0.48721	0.50097	**H192**	0.14443	0.04449	1.08907
**H89**	0.56061	-0.19023	0.30939	**H193**	0.69624	0.92625	0.96388
**H90**	1.47762	-0.14487	0.44558	**H194**	0.64066	0.02811	0.97913
**H91**	1.00684	0.39824	0.51548	**H195**	0.05424	0.31783	0.7711
**H92**	0.92257	0.43372	0.55121	**H196**	0.97346	0.36168	0.91528
**H93**	0.99322	-0.42727	0.65425	**H197**	0.50858	0.90883	0.93258
**H94**	1.07723	-0.4628	0.61775	**H198**	0.42687	0.92712	1.0141
**H95**	0.58887	-0.00894	0.39092	**H199**	0.49718	0.04787	1.18699
**H96**	0.55467	0.07544	0.42439	**H200**	0.5794	0.0285	1.10778
**H97**	1.40316	0.01212	0.45264	**H201**	0.07673	0.49834	0.85593
**H98**	1.43709	-0.07183	0.41589	**H202**	0.04142	0.58167	0.89416
**H99**	1.42912	0.19127	0.38562	**H203**	0.88975	0.51716	0.90608
**H100**	0.52179	0.14687	0.49624	**H204**	0.9256	0.43345	0.86559
**H101**	0.81672	-0.42778	0.55298	**H205**	0.91988	0.69927	0.86029
**H102**	0.84835	0.46427	0.60937	**H206**	0.00736	0.6493	0.97795
**C103**	0.0927	0.90746	0.98508	**H207**	0.3128	0.01735	1.23311
**C104**	0.10869	0.95851	0.99297	**H208**	0.3546	0.97085	1.03649

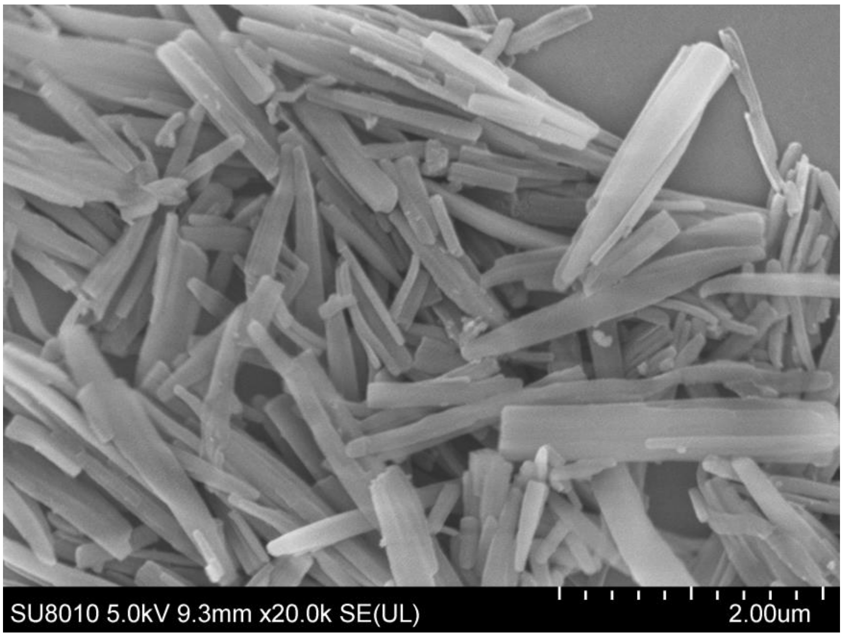

Supplementary Fig. 8 | SEM image of Py-DAB.

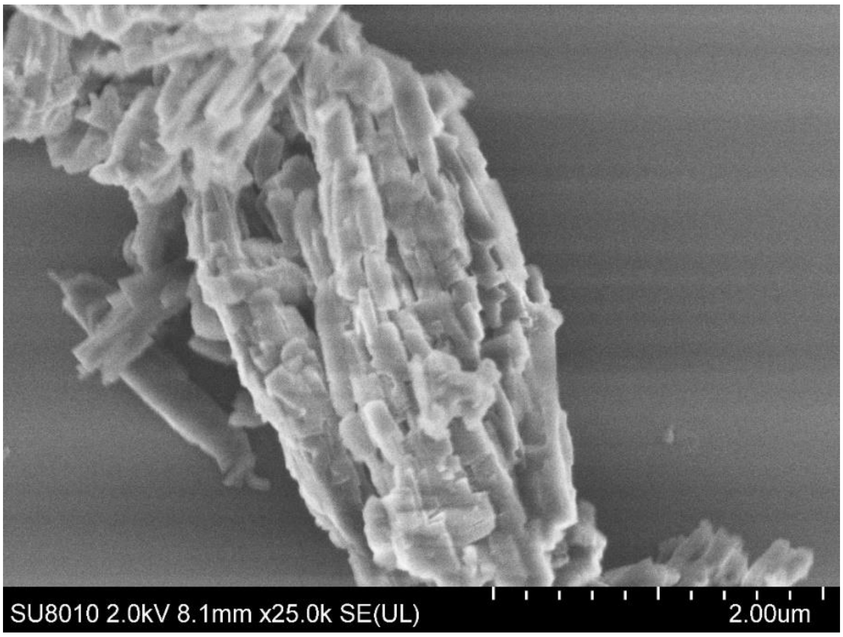

Supplementary Fig. 9 | SEM image of PyN-DAB.

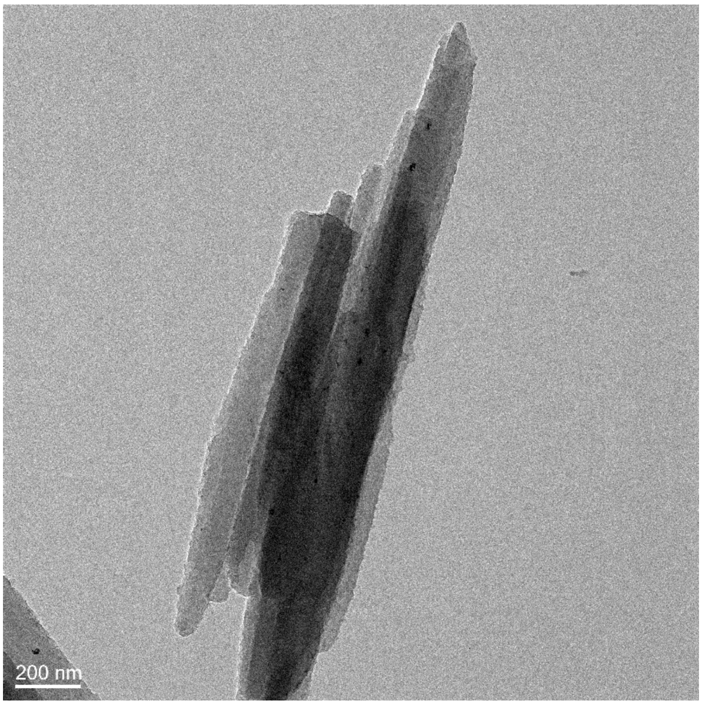

Supplementary Fig. 10 | HR-TEM image of Py-DAB.

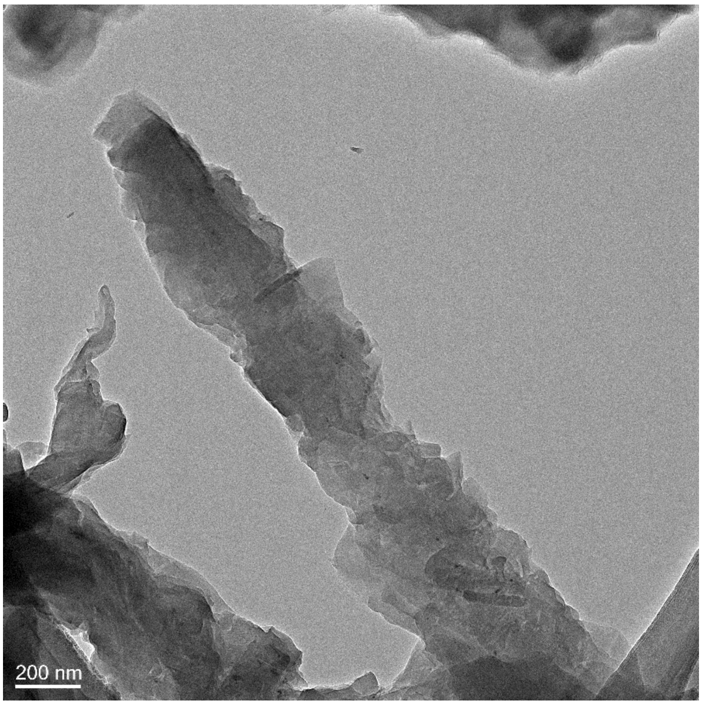

Supplementary Fig. 11 | HR-TEM image of PyN-DAB.

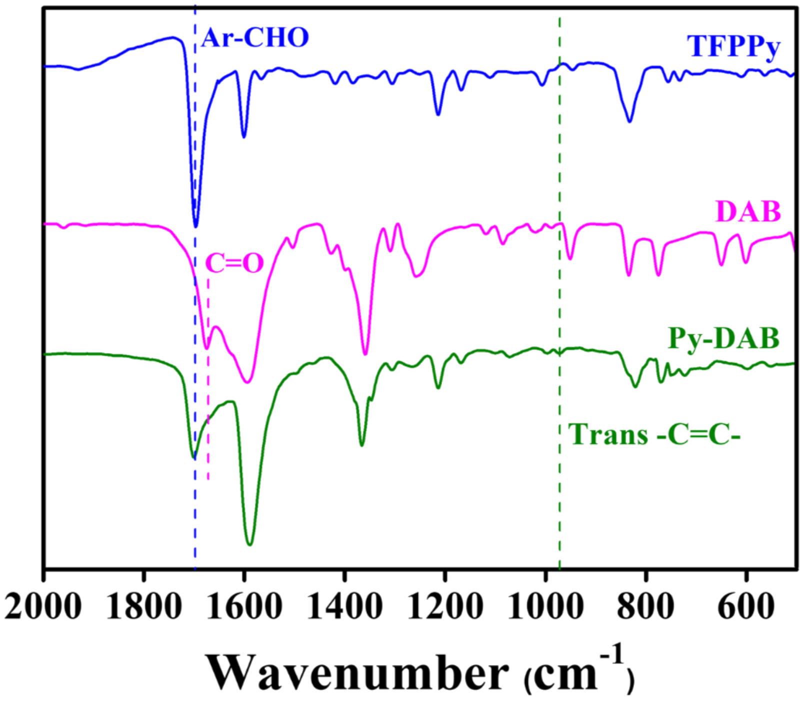

Supplementary Fig. 12 | FT-IR spectra of TFPPy, DAB, and Py-DAB.

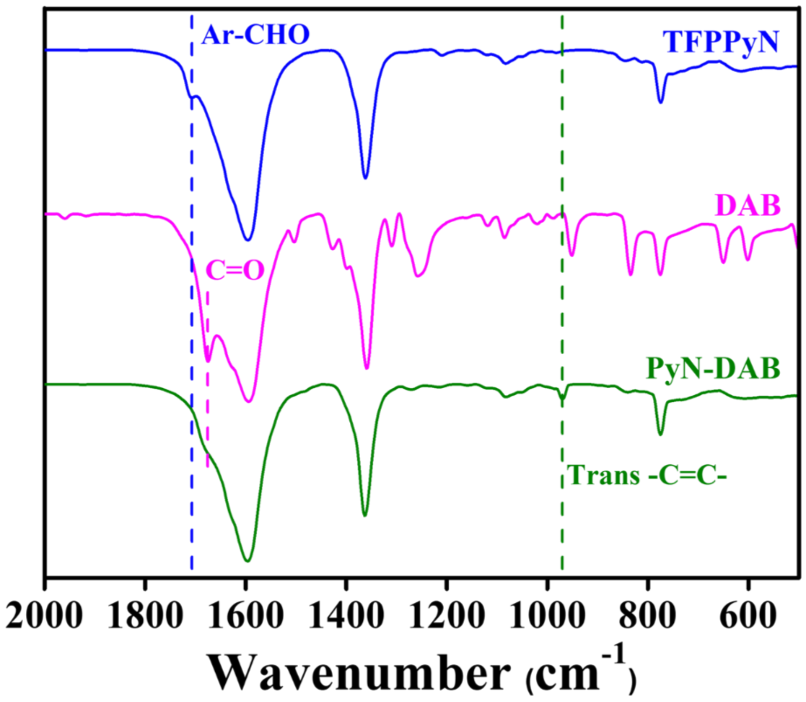

Supplementary Fig. 13 | FT-IR spectra of TFPPyN, DAB, and PyN-DAB.

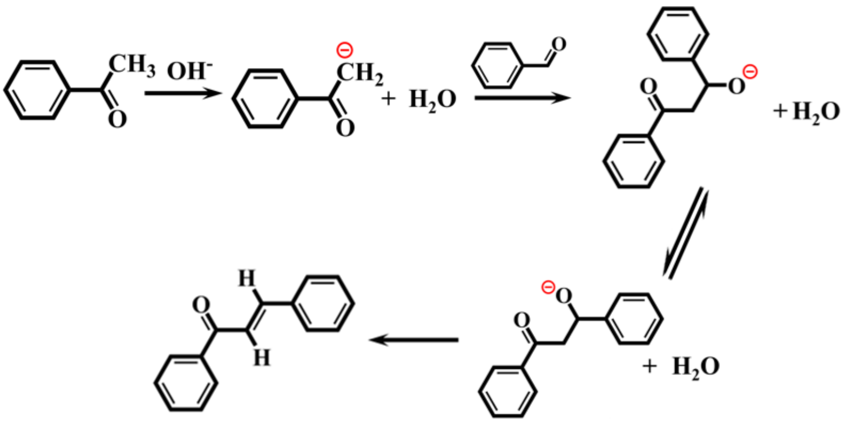

Supplementary Fig. 14 | Scheme of proposed mechanism of the trans C=C formation of model reaction using Claisen-Schmidt reaction under catalyst of NaOH.

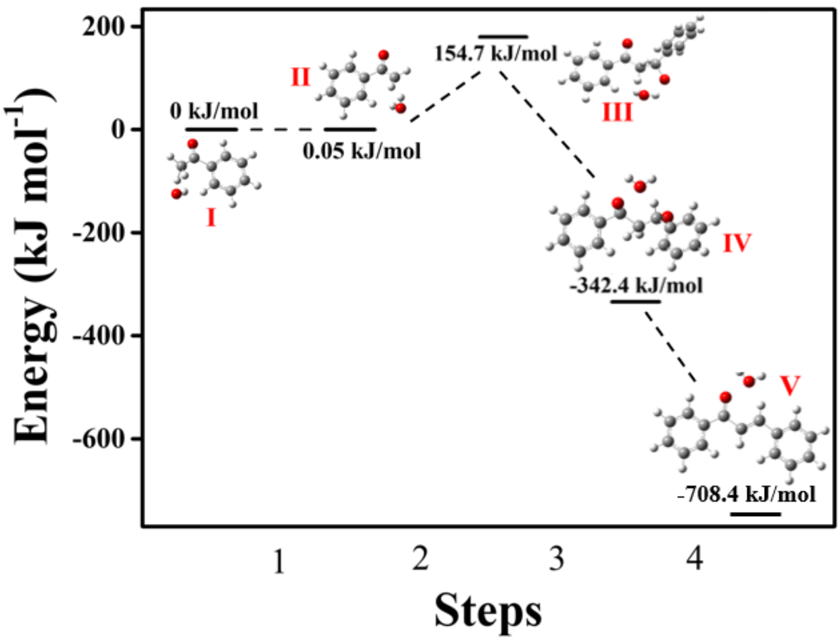

Supplementary Fig. 15 | DFT calculated molecular conformation and energy profiles of the suggested Claisen-Schmidt leading to the trans -C=C- bond under catalysis of NaOH.

In step 1, the 1,4-diacetophenone I tends to be easily deprotonated by OH^- to form the relatively low energy carbon anion species II (E=0.05 kJ/mol). Afterward, the carbanion species II attacks the aldehyde to yield the intermediate III (E=154.7 kJ/mol). Subsequent formation of an intermediate IV (E=-342.4 kJ/mol) due to thermodynamic advantages and the irreversible elimination of stable result in the trans-vinylene (E=-708.4 kJ/mol).

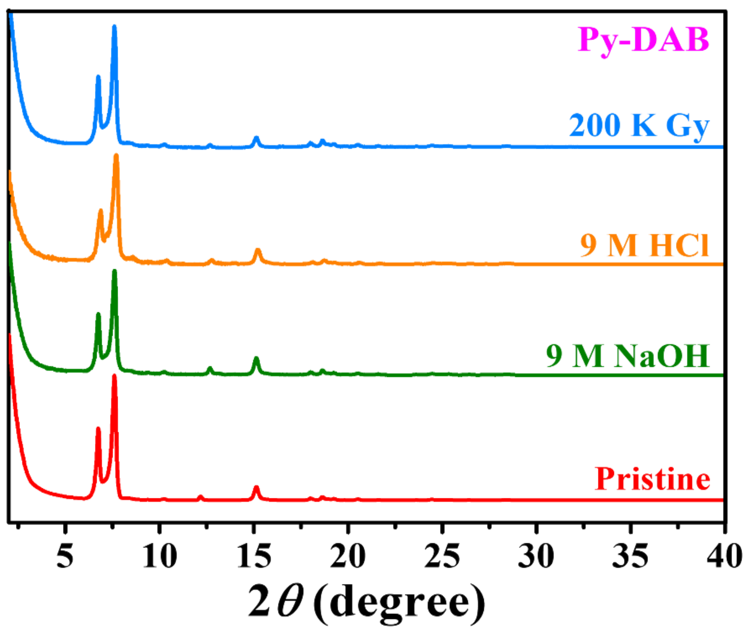

Supplementary Fig. 16 | PXRD spectra of Py-DAB treated for 24 h under different conditions, included in NaOH (9.0 M), HCl (9.0 M) and 200K Gy.

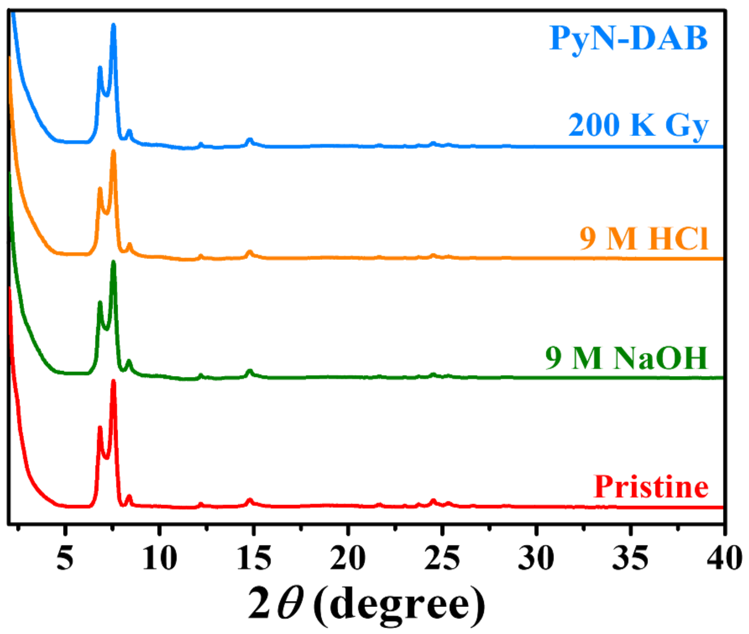

Supplementary Fig. 17 | PXRD spectra of PyN-DAB treated for 24 h under different conditions, included in NaOH (9.0 M), HCl (9.0 M) and 200K Gy.

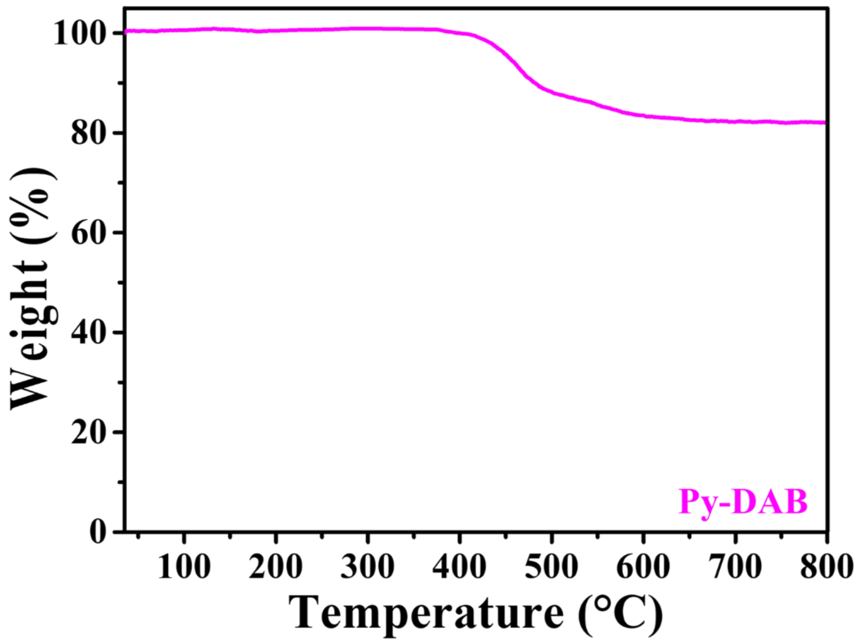

Supplementary Fig. 18 | TGA data indicates that Py-DAB is thermally stable up to *ca*. 425 ℃.

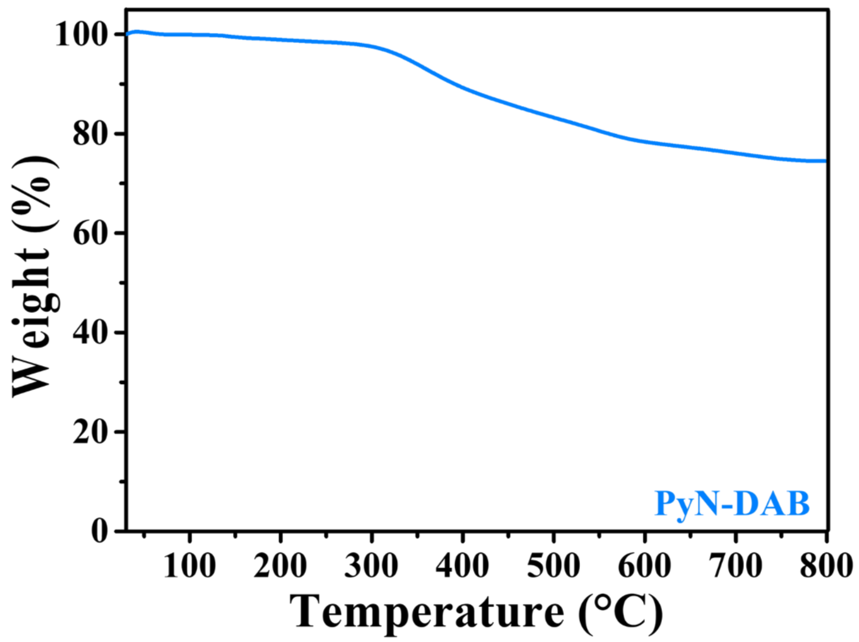

Supplementary Fig. 19 | TGA data indicates that PyN-DAB is thermally stable up to *ca*. 325 ℃.

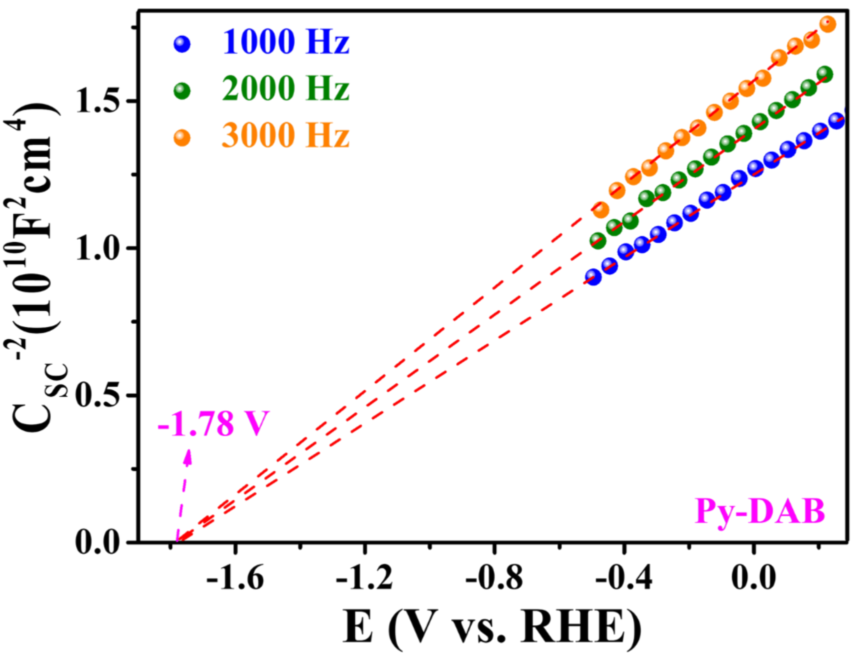

Supplementary Fig. 20 | The relative band positions of Py-DAB can be determined as -1.78 V according to the Mott-Schottky results.

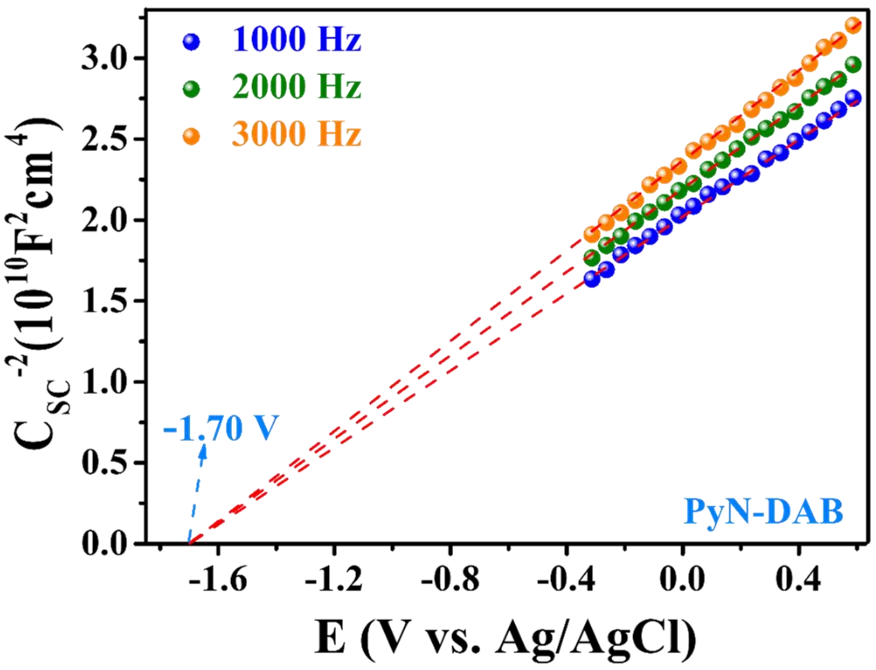

Supplementary Fig. 21 | The relative band positions of PyN-DAB can be determined as -1.70 V according to the Mott-Schottky results.

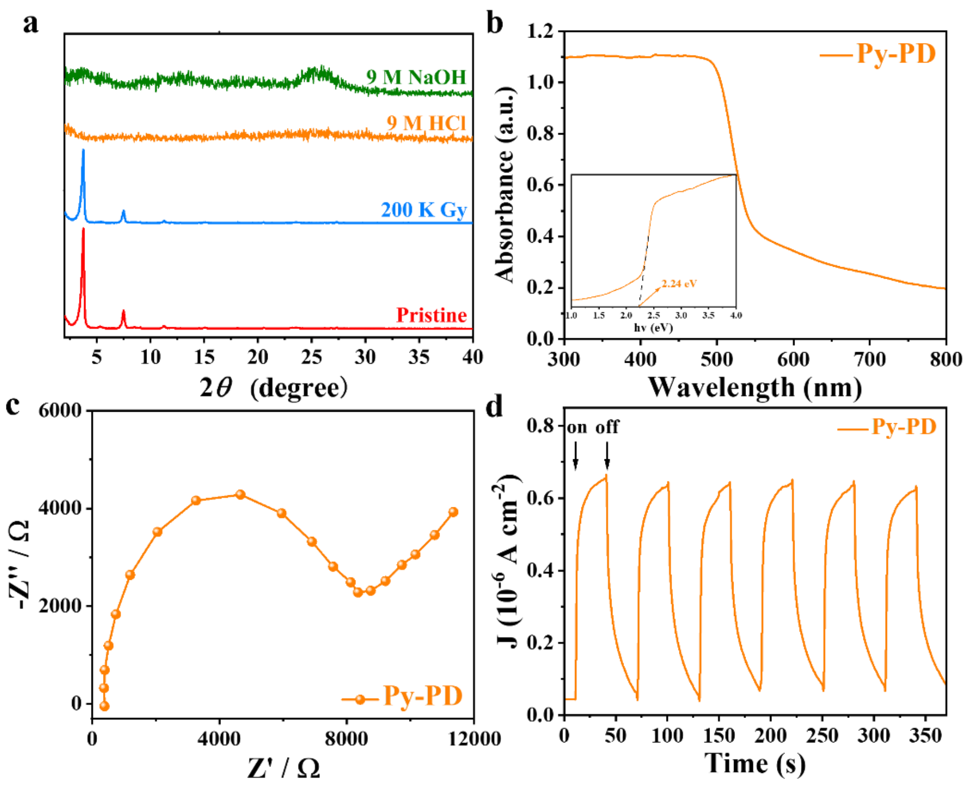

Supplementary Fig. 22 | a PXRD spectra of Py-PD treated for 24 h under different conditions, included in NaOH (9.0 M), HCl (9.0 M) and 200 K Gy. **b** UV-vis diffuse reflection spectrum of Py-PD (Inset: the optical band gaps). **c** EIS curves of Py-PD. **d** Photocurrent generation test spectrum of Py-PD.

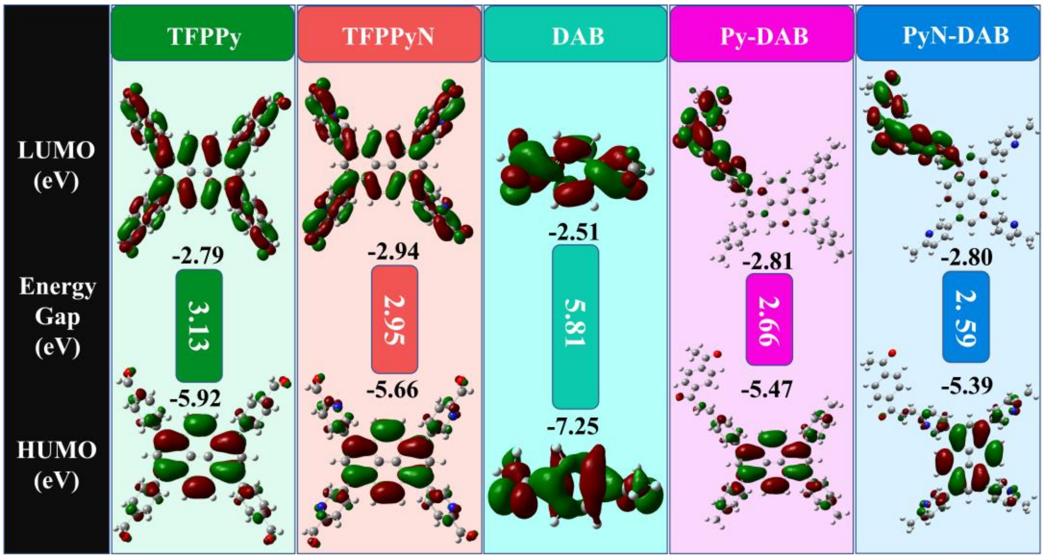

Supplementary Fig. 23 | Calculated spatial distributions of HOMO and LUMO in the model of Py-DAB, PyN-DAB and involved monomers based on the optimized ground-state geometric structures.

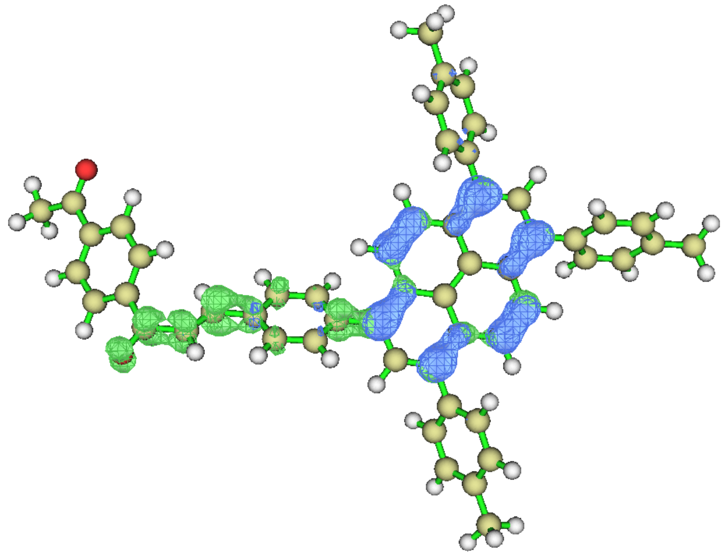

Supplementary Fig. 24 | Theoretical calculations of the separation situation of the electron from the hole in Py-DAB (blocky areas of green represents electrons cloud, blue represents holes), and the distance between the electrons and the holes centroid is 2.526 Å according to the calculation result of D index formula.

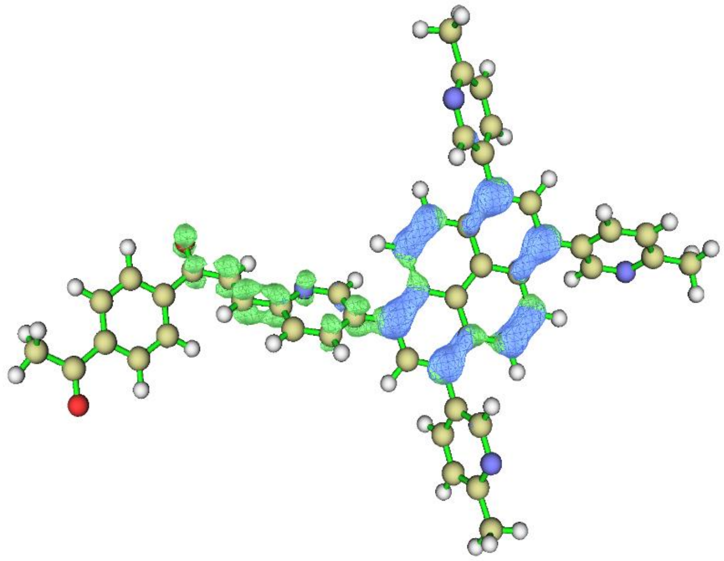

Supplementary Fig. 25 | Theoretical calculations of the separation situation of the electron from the hole in PyN-DAB (blocky areas of green represents electrons cloud, blue represents holes), and the distance between the electrons and the holes centroid is 1.998 Å according to the calculation result of D index formula.

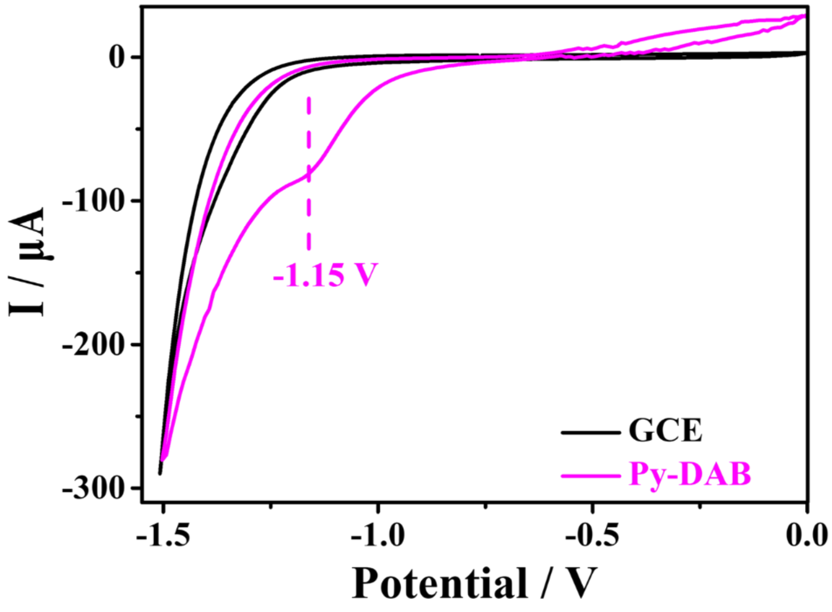

Supplementary Fig. 26 | Corresponding CVs of GCE and Py-DAB.

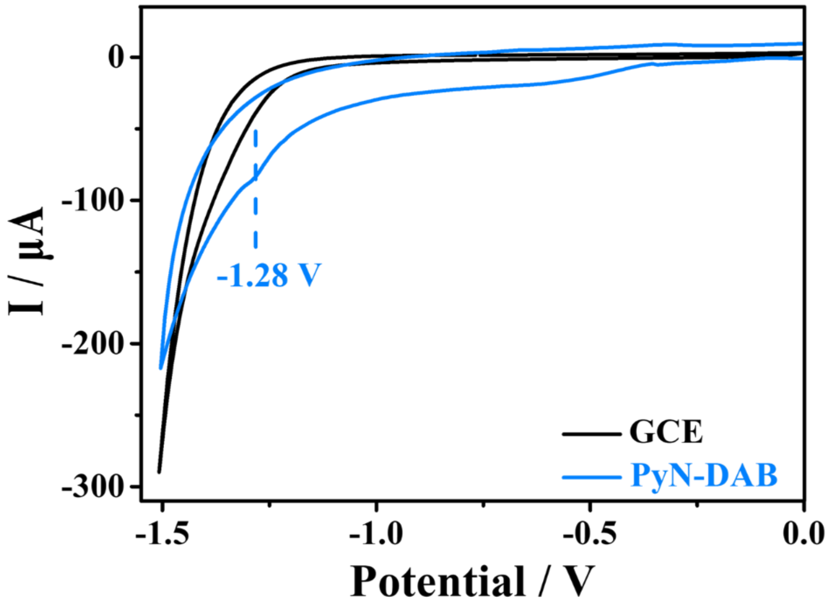

Supplementary Fig. 27 | Corresponding CVs of GCE and PyN-DAB.

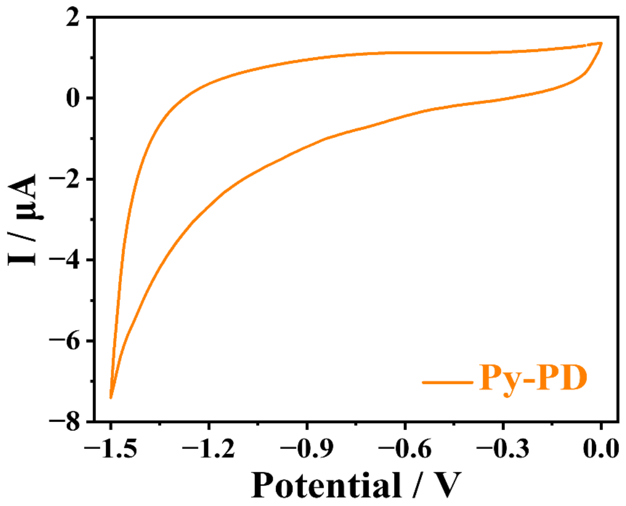

Supplementary Fig. 28 | CV curve of Py-PD.

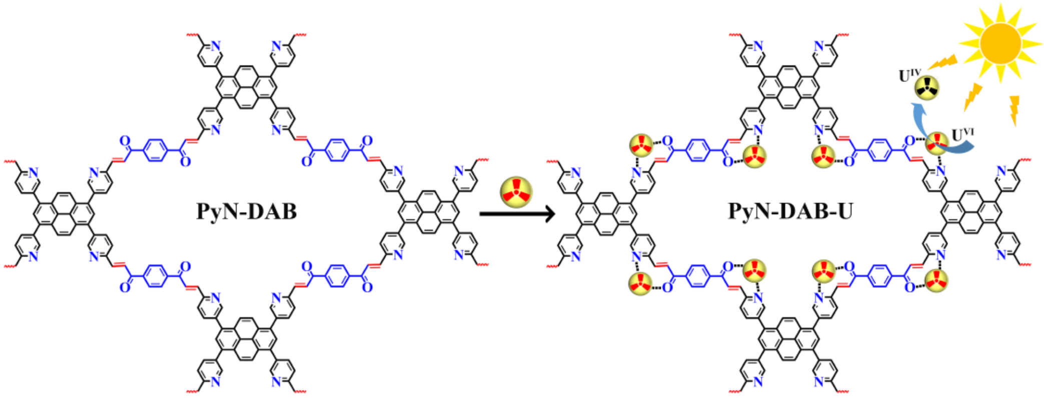

Supplementary Fig. 29 | Process diagram of PyN-DAB for photocatalytic reduction of UO_2^{2+}.

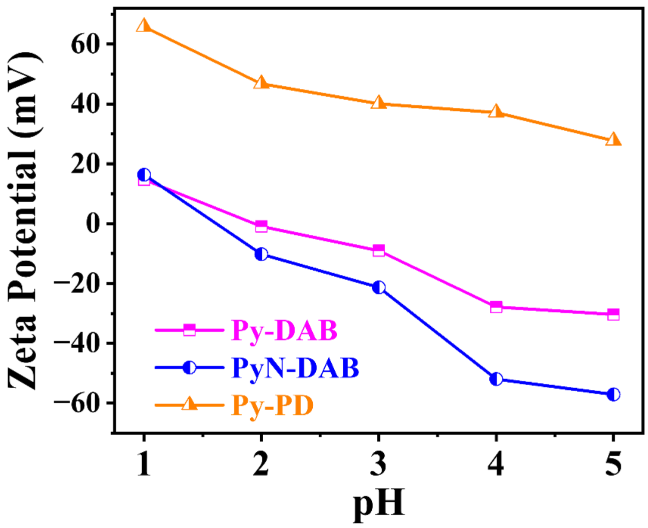

Supplementary Fig. 30 | The Zeta potential of Py DAB, PyN DAB and Py-PD.

Supplementary Table 5 | Comparison of the UO_2^{2+} sorption capacity of PyN-DAB with other sorbents.

Absorbents	Adsorption capacity (mg g^{-1})	Ref.
Mesoporous Carbon Materials	97.0	1
MIL-125-P@TiO_2	614.8	2
GO-pDA-PEI	530.6	3
Fe_3O_4@ZIF-8	523.5	4
MOF-76	298	5
PPAFs	147.6	6
CMPAO	251.9	7
COF-TpDb-AO	408	8
PyN-DAB	1436.4	This work

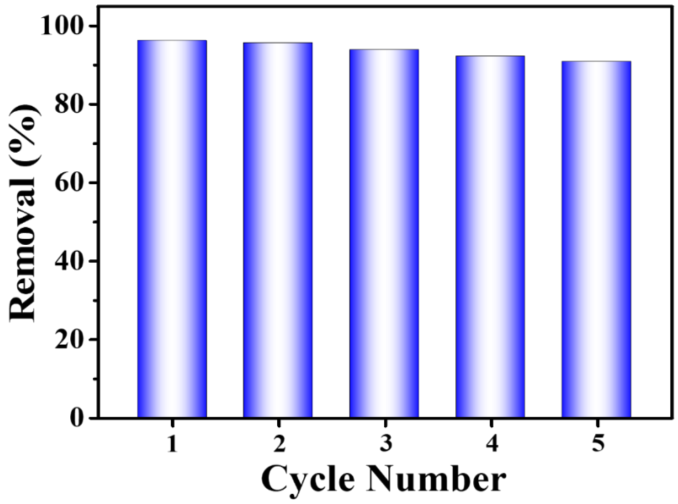

Supplementary Fig. 31 | Recycle use of PyN-DAB for removal of UO_2^{2+} in simulated nuclear industry wastewater.

After treating with the 0.1M HNO_3 aqueous solution,[9] PyN-DAB was easily recovered and could be repeatedly used, a little decrease in uranium removal rate may be attributed to a part of uranium combined closely with PyN-DAB was not eluted thoroughly. The elution mechanism is that the concentration of H^+ in the elution solution is much higher than the adsorption experiment, a large amount of H^+ will occupy the binding sites of uranyl on COF during the desorption process, resulting in the successful desorption of uranyl from COF.

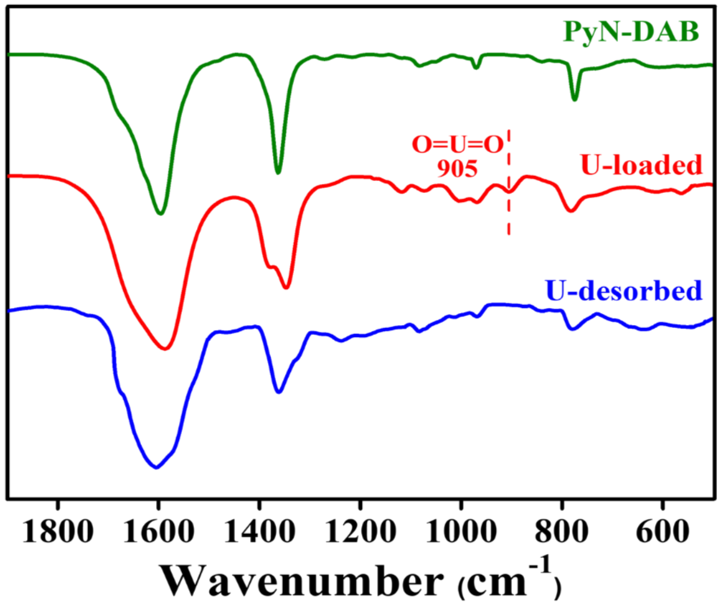

Supplementary Fig. 32 | FTIR spectra of PyN-DAB before, U-loaded and U-desorbed.

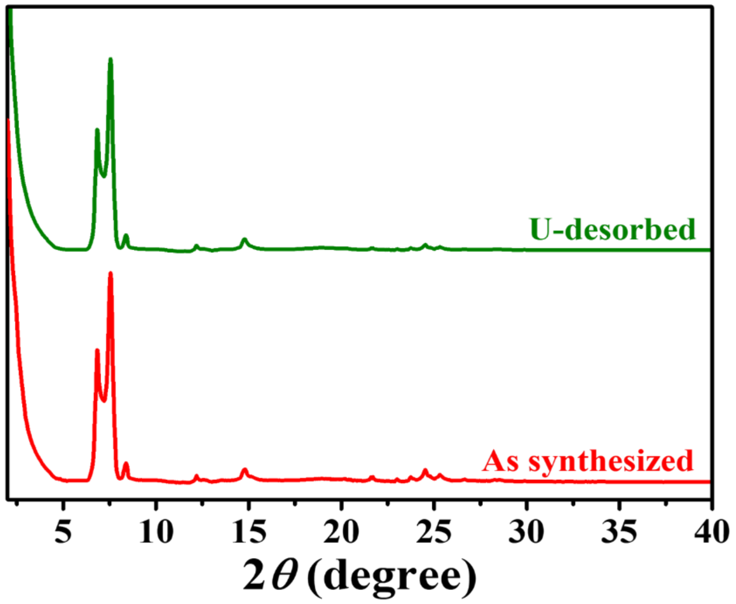

Supplementary Fig. 33 | PXRD spectra of PyN-DAB and U-desorbed.

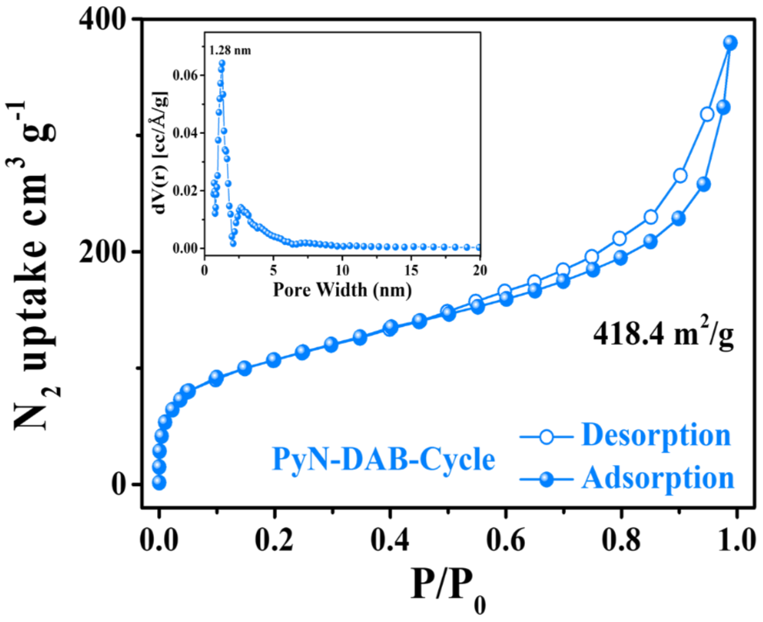

Supplementary Fig. 34 | N_2 sorption isotherms of PyN-DAB after U-desorption (Insets: pore size distributions centered at 1.28 nm), which was barely changed than as-synthesized PyN-DAB (about 456.3 m^2 g^{-1}).

Supplementary Table 6 | Ion composition and concentration in Iranian nuclear industry wastewater.

**Component**	**Concentration (mg L^{-1})**
U	28.76
Ni	22.12
Cu	12.63
Fe	0.01
Mn	0.02
Pb	0.05
Ca	2.74
Mg	0.02

Supplementary Table 7 | Ion composition and concentration of Sample 1 in uranium mine wastewater.

**Component of Sample 1**	**Concentration (pH=1.7)**
U	0.421 mg L^{-1}
Cd	0.00023 mg L^{-1}
Pb	0.00057 mg L^{-1}
^{226}Ra	0.131 Bq L^{-1}
^{210}Po	0.014 Bq L^{-1}
^{210}Pb	0.03 Bq L^{-1}

Supplementary Table 8 | Ion composition and concentration of Sample 2 in uranium mine wastewater.

**Component of Sample 2**	**Concentration (pH=1.6)**
U	0.0256 mg L^{-1}
Cd	0.00072 mg L^{-1}
Pb	0.00096 mg L^{-1}
Mn	0.647 mg L^{-1}
^{226}Ra	0.031 Bq L^{-1}
^{210}Po	0.003 Bq L^{-1}

Supplementary Table 9 | Ion composition and concentration of Sample 3 in uranium mine wastewater.

**Component of Sample 3**	**Concentration (pH=3.1)**
U	0.878 mg L^{-1}
Cd	0.297 mg L^{-1}
Zn	3.460 mg L^{-1}
As	0.003 mg L^{-1}
Ni	1.65 mg L^{-1}
^{226}Ra	0.269 Bq L^{-1}
^{210}Po	0.004 Bq L^{-1}

Supplementary Table 10 | Ion composition and concentration of Sample 4 in uranium mine wastewater.

Component of Sample 4	Concentration (pH=8.0)
U	0.237 mg L^{-1}
Cd	0.00396 mg L^{-1}
Pb	0.00037 mg L^{-1}
^{226}Ra	0.118 Bq L^{-1}
^{210}Po	0.005 Bq L^{-1}

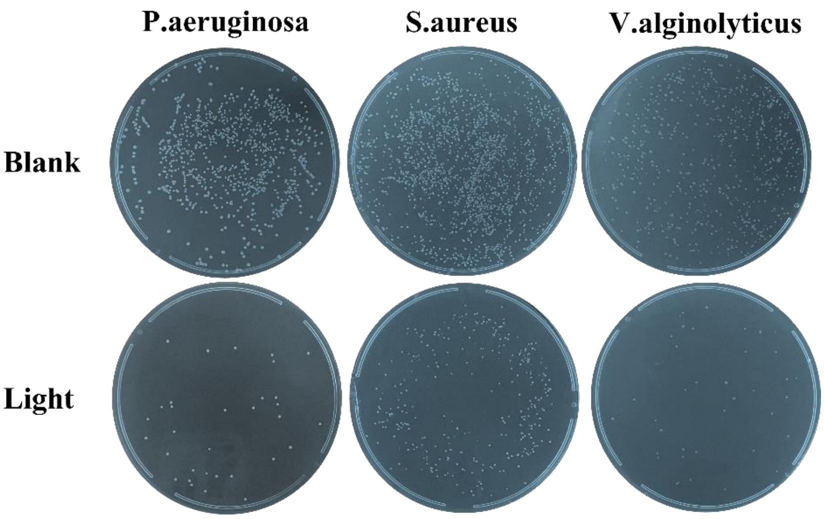

Supplementary Fig. 35 | Antibacterial activity of PyN-DAB under the presence of *P. aeruginosa*, *S. aureus* and *V. alginolyticus*.

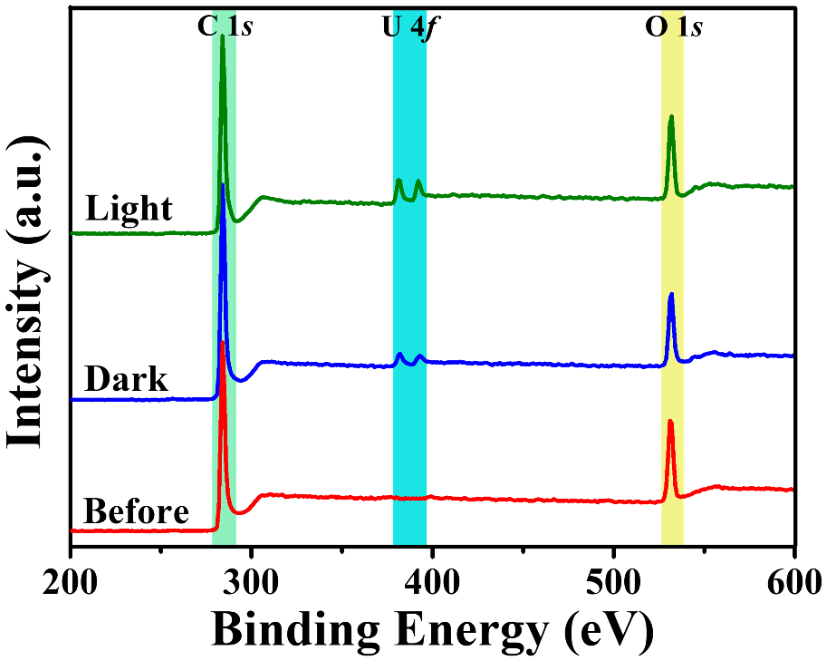

Supplementary Fig. 36 | XPS survey spectra of Py-DAB measured before and after loaded with uranium in the dark or under visible light irradiation.

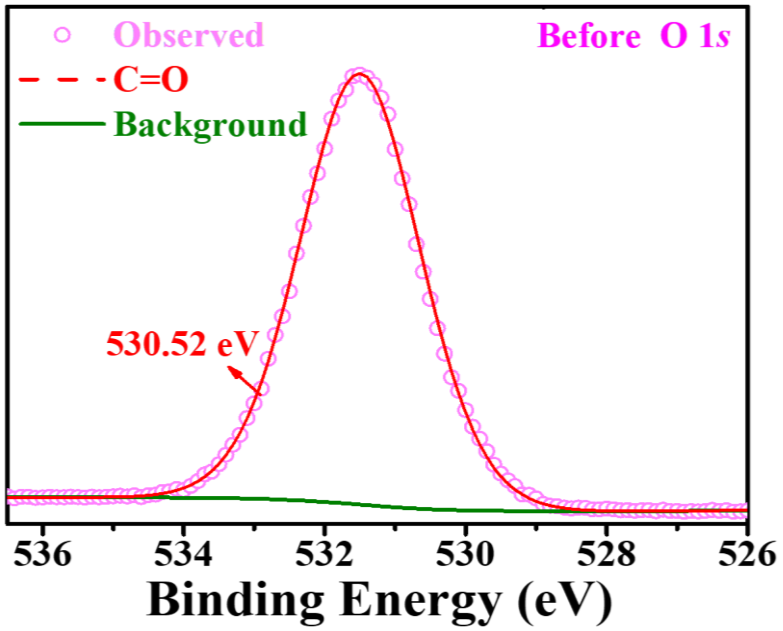

Supplementary Fig. 37 | High-resolution XPS spectra of O 1*s* for Py-DAB, the peaks of 530.52 eV belonged to C=O.

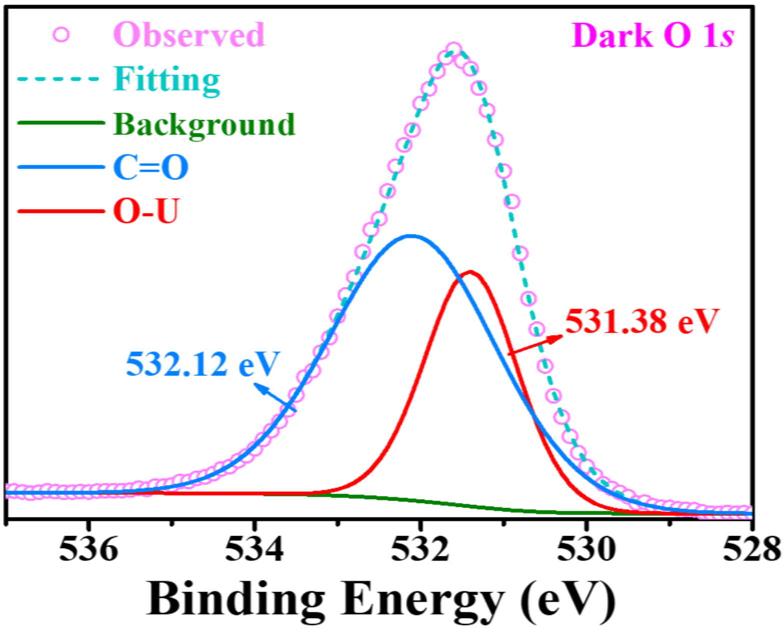

Supplementary Fig. 38 | High-resolution XPS spectra of O 1*s* for Py-DAB loaded with uranium in the dark, the peaks of 531.38 eV and 532.12 eV belonged to O-U and C=O, respectively.

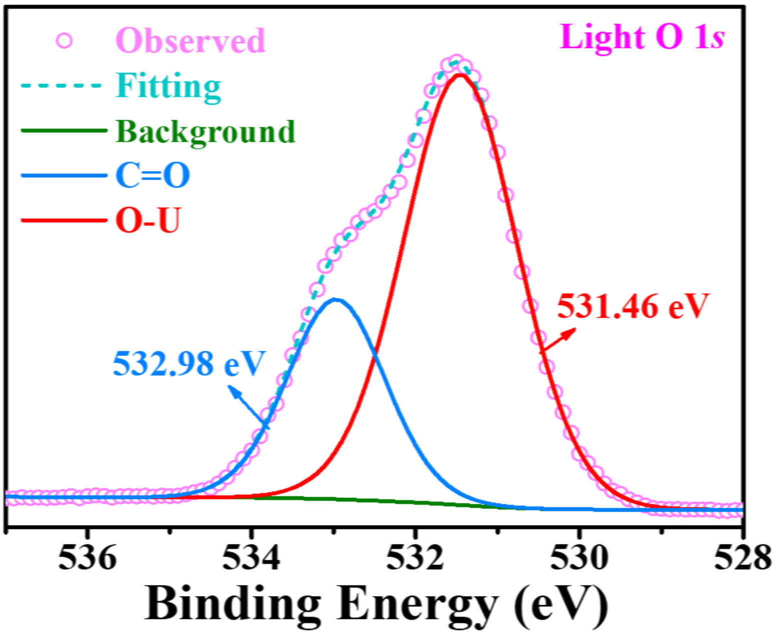

Supplementary Fig. 39 | High-resolution XPS spectra of O 1*s* for Py-DAB loaded with uranium in the light, the peaks of 531.46 eV and 532.98 eV belonged to O-U and C=O, respectively.

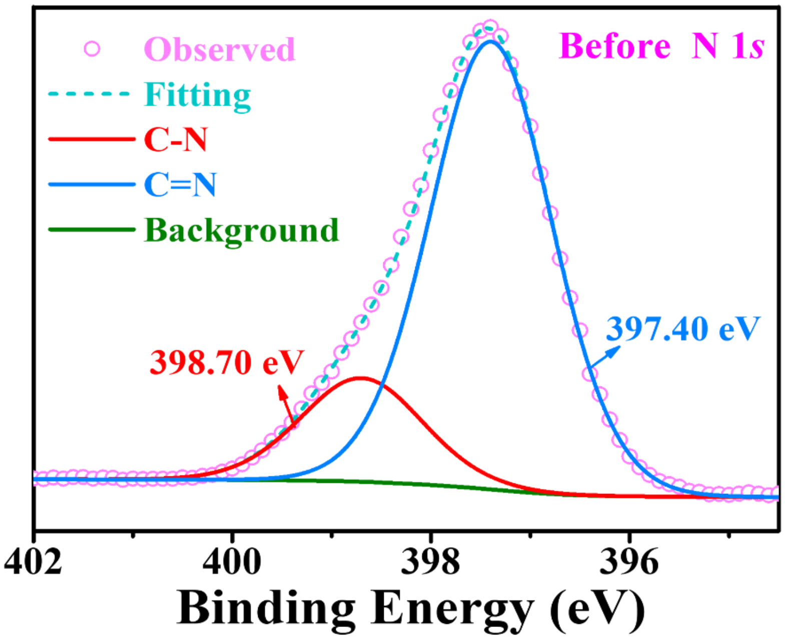

Supplementary Fig. 40 | High-resolution XPS spectra of N 1*s* for PyN-DAB, the peaks of 397.40 eV and 398.70 eV belonged to C=N and C-N, respectively.

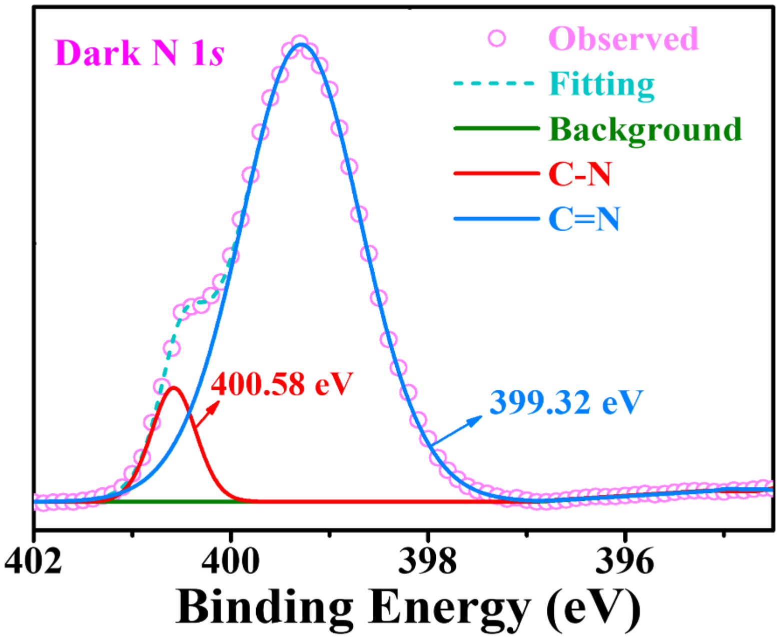

Supplementary Fig. 41 | High-resolution XPS spectra of N 1*s* for PyN-DAB loaded with uranium in the dark, the peaks of 399.32 eV and 400.58 eV belonged to C=N and C-N, respectively.

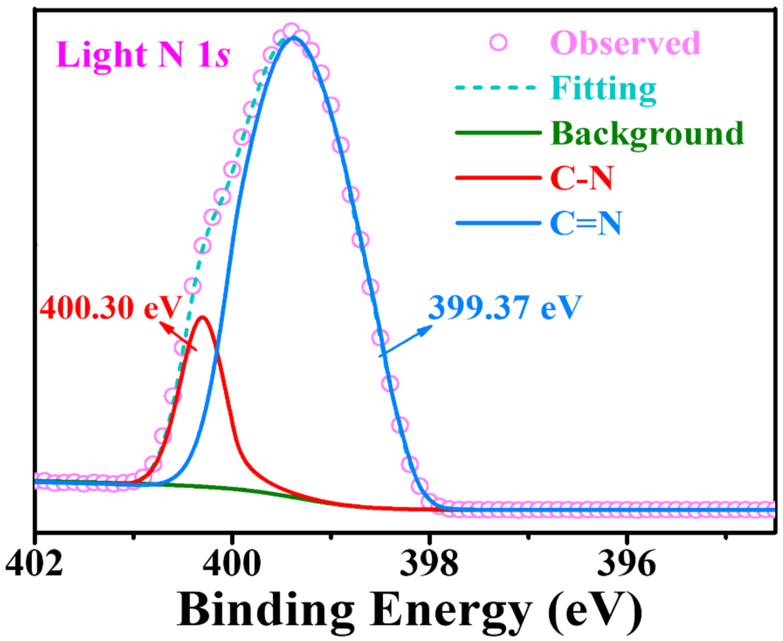

Supplementary Fig. 42 | High-resolution XPS spectra of N 1*s* for PyN-DAB loaded with uranium under visible light irradiation, the peaks of 399.37 eV and 400.30 eV belonged to C=N and C-N, respectively.

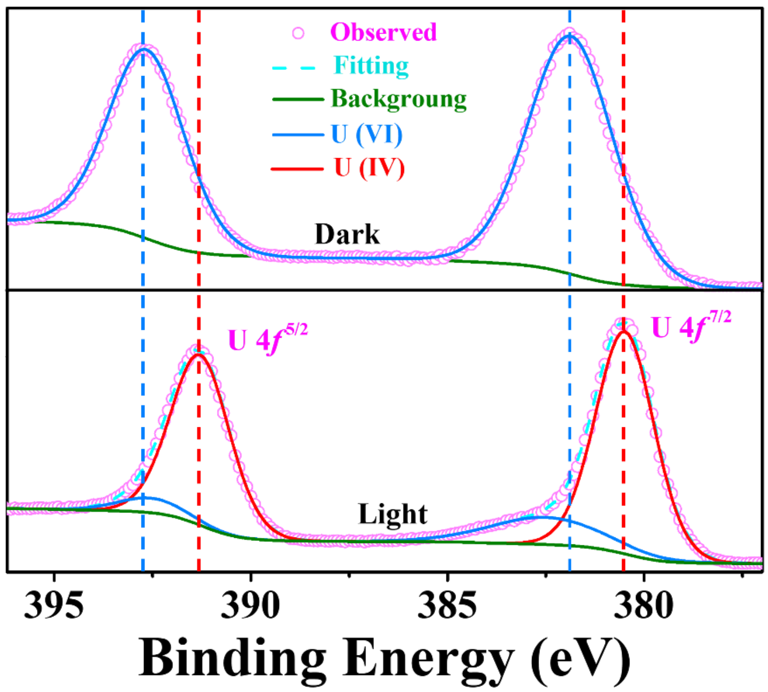

Supplementary Fig. 43 | U 4*f* high-resolution spectra of Py-DAB loaded with uranium in the dark or under visible light irradiation.

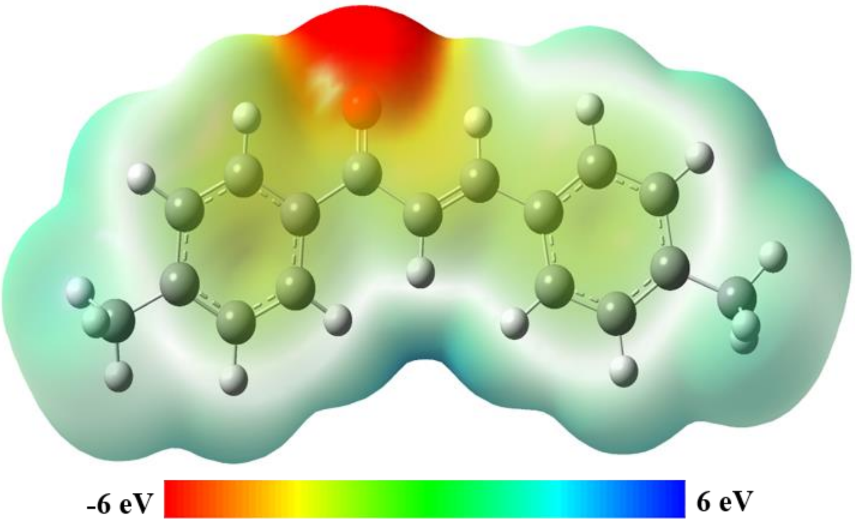

Supplementary Fig. 44 | Corresponding electrostatic surface potential distribution of C.

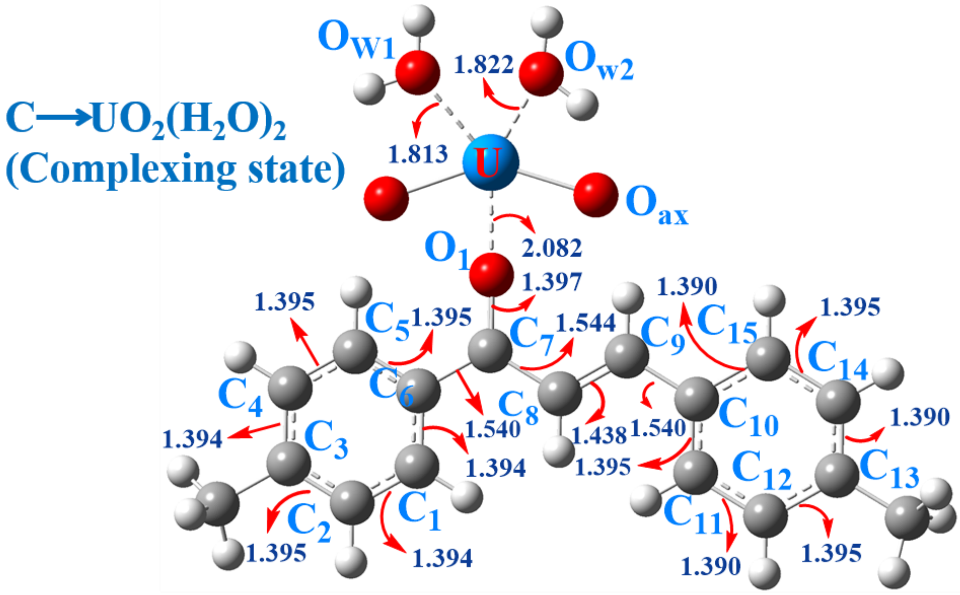

Supplementary Fig. 45 | The optimized structures for the complexing state of uranium with C, and the corresponding energy is -107.4 kJ mol^{-1}.

4. Supplementary References.

[1] Carboni, M., Abney, C. W., Taylor-Pashow, K. M. L., Vivero-Escoto, J. L. & Lin, W. Uranium sorption with functionalized mesoporous carbon materials. *Ind. Eng. Chem. Res.* **52**, 15187-15197 (2013).

[2] Xu, C. et al. Synthesis of NH_2-MIL-125/NH_2-MIL-125-P@TiO_2 and its adsorption to uranyl ions. *ChemistrySelect* **4**, 12801-12806 (2019).

[3] Li, S. et al. Graphene oxide based dopamine mussel-like cross-linked polyethylene imine nanocomposite coating with enhanced hexavalent uranium adsorption. *J, Mater, Chem, A* **7**, 16902-16911 (2019).

[4] Min, X. et al. Fe_3O_4@ZIF-8: a magnetic nanocomposite for highly efficient UO_2^{2+} adsorption and selective UO_2^{2+}/Ln^{3+} separation. *Chem. Commun.* **53**, 4199-4202 (2017).

[5] Yang, W. et al. MOF-76: from a luminescent probe to highly efficient U(VI) sorption material. *Chem. Commun.* **49**, 10415-10417 (2013).

[6] Wang, T., Xu, M., Han, X., Yang, S. & Hua, D. Petroleum pitch-based porous aromatic frameworks with phosphonate ligand for efficient separation of uranium from radioactive effluents. *J. Hazard. Mater.* **368**, 214-220 (2019).

[7] Xu, M. et al. Highly fluorescent conjugated microporous polymers for concurrent adsorption and detection of uranium. *J, Mater, Chem, A* **7**, 11214-11222 (2019).

[8] Sun, Q. et al. Covalent organic frameworks as a decorating platform for utilization and affinity enhancement of chelating sites for radionuclide sequestration. *Adv. Mater.* **30**, 1705479 (2018).

[9] Cui, W.-R. et al. Low band gap benzoxazole-linked covalent organic frameworks for photo-enhanced targeted uranium recovery. *Small* **17**, 2006882 (2021).
